# A Rac/Cdc42 exchange factor complex promotes formation of lateral filopodia and blood vessel lumen morphogenesis

**DOI:** 10.1038/ncomms8286

**Published:** 2015-07-01

**Authors:** Sabu Abraham, Margherita Scarcia, Richard D. Bagshaw, Kathryn McMahon, Gary Grant, Tracey Harvey, Maggie Yeo, Filomena O.G. Esteves, Helene H. Thygesen, Pamela F. Jones, Valerie Speirs, Andrew M. Hanby, Peter J. Selby, Mihaela Lorger, T. Neil Dear, Tony Pawson, Christopher J. Marshall, Georgia Mavria

**Affiliations:** 1 Institute of Cancer Research, Division of Cancer Biology, 237 Fulham Road, London SW3 6JB, UK; 2 Leeds Institute of Cancer and Pathology, University of Leeds, St James' University Hospital, Beckett Street, Leeds LS9 7TF, UK; 3 Samuel Lunenfeld Research Institute, Mount Sinai Hospital, 600 University Avenue, Toronto, M5G 1X5 Ontario, Canada; 4 Leeds Institutes of Molecular Medicine, University of Leeds, St James' University Hospital, Beckett Street, Leeds LS9 7TF, UK

## Abstract

During angiogenesis, Rho-GTPases influence endothelial cell migration and cell–cell adhesion; however it is not known whether they control formation of vessel lumens, which are essential for blood flow. Here, using an organotypic system that recapitulates distinct stages of VEGF-dependent angiogenesis, we show that lumen formation requires early cytoskeletal remodelling and lateral cell–cell contacts, mediated through the RAC1 guanine nucleotide exchange factor (GEF) DOCK4 (dedicator of cytokinesis 4). DOCK4 signalling is necessary for lateral filopodial protrusions and tubule remodelling prior to lumen formation, whereas proximal, tip filopodia persist in the absence of DOCK4. VEGF-dependent Rac activation via DOCK4 is necessary for CDC42 activation to signal filopodia formation and depends on the activation of RHOG through the RHOG GEF, SGEF. VEGF promotes interaction of DOCK4 with the CDC42 GEF DOCK9. These studies identify a novel Rho-family GTPase activation cascade for the formation of endothelial cell filopodial protrusions necessary for tubule remodelling, thereby influencing subsequent stages of lumen morphogenesis.

## Abstract (web summary)

Blood vessel development depends upon endothelial cell migration and adhesion, which are regulated by Rho-GTPases. Here the authors identify Rho-GTPase guanine nucleotide exchange factors that specifically control lateral filopodial contacts and are required for lumen formation during angiogenesis.

## Graphical abstract

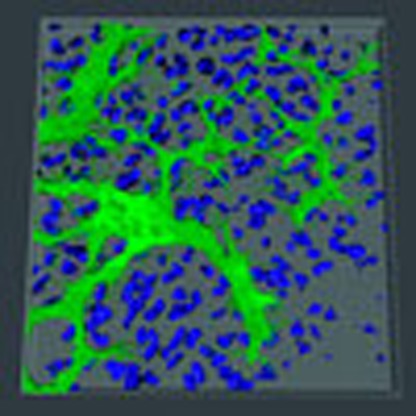

Angiogenesis is the most common mechanism for physiological and pathological vascular expansion from pre-existing blood vessels. In cancer, high levels of the angiogenic factor VEGF secreted by tumour and stromal cells drives aberrant angiogenic growth that results in tumour blood vessel tortuosity and hypoxia^1^,^2^. Vessel growth from pre-existing vessels can be through elongation or branching, both of which require migration of a leading cell that drives the collective migration of adjacent endothelial cells, a process known as sprouting. Sprouting may be proximal, promoting elongation or lateral resulting in branches^3^. Lateral sprouting requires localized weakening of intercellular junctions to allow an endothelial cell to break away from the vessel wall and become a leading cell capable of forming a new sprout^4^. Sprouting is preceded by acquisition of endothelial tip cell morphology characterized by the extension of numerous filopodial protrusions^5^,^6^. Filopodia on tip cells are able to sense the surrounding microenvironment and drive rapid extension of a sprout for correct patterning of the developing blood vessels^5^,^7^. In addition, filopodia can initiate intercellular contacts and bridging of endothelial cells through trafficking and presentation of the adherens junction molecule VE-cadherin^7^,^8^. Following expansion, blood vessel functionality requires organization of the endothelial network into three-dimensional (3D) tubular structures with lumens^9^.

Signalling to the actin cytoskeleton is central to growth factor signalling during angiogenesis^10^,^11^. The actin cytoskeleton and cellular processes dependent on it are regulated through the activity of Rho-GTPases that cycle between active GTP- and inactive GDP-bound states, controlled by positive regulators and negative regulators (guanine nucleotide exchange factors (GEFs) and GTPase-activating proteins, respectively). Endothelial cell motility and VEGF-driven migration require Rac1 and RhoJ^12^,^13^, cell assembly *in vivo* depends on activation of RhoA^14^, while suppression of sprouting through adherens junctions requires RhoC signalling to Rho-kinases and actomyosin contractility^4^. To identify Rho GEFs required for distinct processes in angiogenesis, we performed short interfering RNA (siRNA)-based screens in an organotypic angiogenesis system that recapitulates distinct stages of endothelial cell association, sprouting and tubule establishment^4^,^15^. These screens identified the Rac1 GEF DOCK4, a member of the DOCK (dedicator of cytokinesis) 180 family GEFs^16^, as key regulator of filopodia formation and angiogenesis. Previous studies have shown that DOCK4 controls neuronal outgrowth and branching^17^, breast cancer cell and fibroblast migration^18^,^19^. Our studies show that through interaction with the Cdc42 GEF DOCK9, DOCK4 controls generation of endothelial cell filopodial protrusions necessary for the dynamic remodelling of tubules, lateral organization of endothelial cells and lumen morphogenesis.

## Results

### DOCK4 controls tubule remodelling and lumen formation

To investigate which GEFs are required for angiogenesis, we modified a coculture angiogenesis assay^15^,^20^ to facilitate the formation of mature tubes with lumens through distinct stages of tubule morphogenesis (Supplementary Fig. 1a and Supplementary Movie 1). Endothelial cells were seeded onto a confluent layer of fibroblasts (CFs); after 14 days culture lumens are present as shown by 3D reconstructions of confocal images (Supplementary Movie 1). Lumens in the coculture assay are multicellular^20^ and form at sites of lateral endothelial cell–cell contacts (Supplementary Fig. 1b–d). Lumenized tubes measured ∼50% of total tubule length and the glycoprotein podocalyxin was localized at the apical side of the lumens as previously described *in vivo*^21^ (Supplementary Fig. 1c). Prior to seeding on CFs, endothelial cells were transfected with RNA interferences (RNAis) targeting RHO-family GEFs. Knockdown of 22 out of 83 tested GEFs^22^ impacted on tubule formation (Supplementary Table 1). DOCK4 knockdown showed marked decrease in the number of branches although the overall amount of tubule formation remained unaffected (Supplementary Fig. 1e), suggesting that DOCK4 is involved in a distinct endothelial cell process.

**Figure 1:**
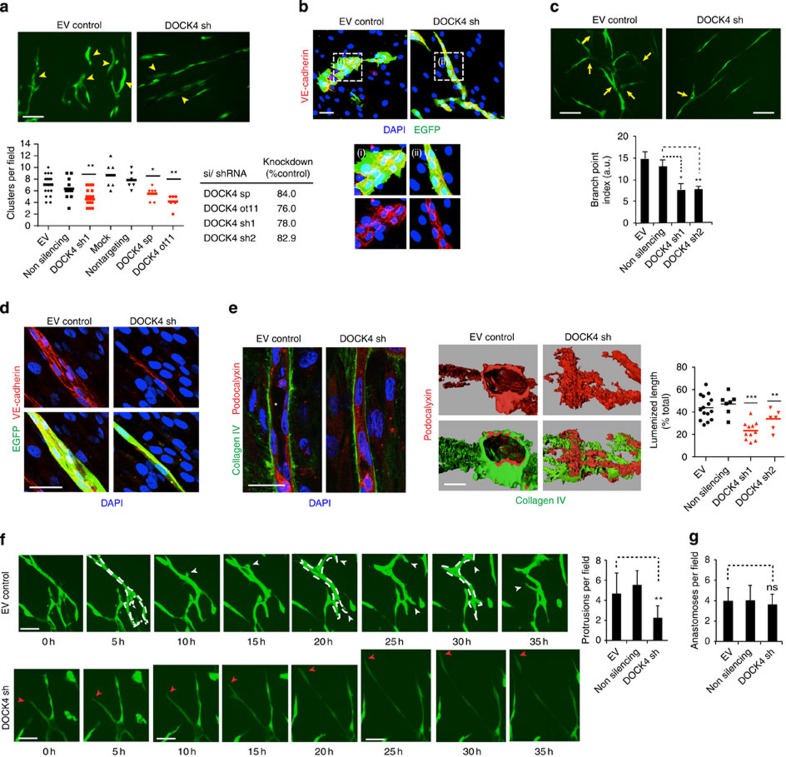
Rac GEF DOCK4 controls lateral remodelling and lumen formation (**a**) Cluster formation (arrowheads) following DOCK4 depletion (EV, empty vector; ot, on-target plus siRNA; sh, shRNA1; sp, smartpool siRNA) in HUVEC 3 days after seeding onto CFs. Scale bar, 100 μm. Scatter plot: cluster quantification, *n*=number of time-lapse movies (EV, *n=*17; non silencing, *n=*12; DOCK4 sh1, *n=*18; mock, *n=*6; nontargeting, *n=*6; DOCK4 sp, *n=*6; DOCK4 ot, *n=*6) from independent experiments (EV, *n=*3; DOCK4 sh1, *n=*3; non silencing, *n=*2; mock, *n=*2; nontargeting, *n=*2; DOCK4 sp, *n=*2; DOCK4 ot, *n=*2); lines represent the mean. Knockdown quantified by quantitative PCR (DOCK4 sp, ot) or immunoblot (DOCK4 sh; shown in Supplementary Fig. 1k). (**b**) VE-cadherin expression following DOCK4 depletion 2 days after seeding HUVEC onto CFs. (i) and (ii): magnifications of outlined boxes. Scale bar, 50 μm. (**c**) Branch formation (arrows) following DOCK4 depletion as in **b** 7 days after seeding HUVEC onto CFs. Scale bar, 100 μm. Histogram: branch point index. For each value, the number of branches divided by tubule length±s.e.m.; *n*=number of organotypic cocultures (EV, *n=*8; non silencing, *n=*11; DOCK4 sh1, *n=*8; DOCK4 sh2, *n=*11) from independent experiments (EV, *n=*3; non silencing, *n=*4; DOCK4 sh1, *n=*3; DOCK4 sh2, *n=*4). (**d**) VE-cadherin expression 7 days after seeding HUVEC with DOCK4 depletion as in **b**. Scale bar 25 μm. (**e**) Lumen formation (asterisk) following DOCK4 depletion as in **c** 14 days after seeding onto CFs. 3D reconstructions are from confocal Z-stacks (Supplementary Movies 1 and 2). Scale bar, 25 μm. Scatter plot: lumenized length as percentage of total length, *n*=number of organotypic cocultures (DOCK4 sh1, *n=*11; DOCK4 sh2, *n=*6; EV, *n=*15; non silencing, *n=*7) from independent experiments (DOCK4 sh1, *n=*2; DOCK4 sh2, *n=*2; EV, *n=*3; non silencing, *n=*2); lines represent the mean. (**f**) Images from Supplementary Movies 3 and 4 of tubule formation following DOCK4 depletion (shRNA1, sh; EV, empty vector) 4 days after seeding onto CFs. Arrowheads: white, protrusions persisting >5 h; red, elongating tip. Scale bar, 50 μM. Histogram: quantifications over 48 h of protrusions persisting >5 h±s.e.m.; *n*=number of time-lapse movies (DOCK4 sh, *n=*19; non silencing, *n=*11; EV, *n=*11) from independent experiments (DOCK4 sh, *n=*3; non silencing, *n=*2; EV, *n=*2). (**g**) Histogram: quantifications over 48 h of anastomoses±s.e.m.; *n*=number of time-lapse movies (non silencing, *n=*11; DOCK4 sh, *n=*19; EV, *n=*17) from independent experiments (non silencing, *n=*2; DOCK4 sh1, *n=*3; EV, *n=*3); NS, non significant by two-tailed *t*-test. Images from Supplementary Movies 5 and 6 showing anastomoses in Supplementary Fig. 1j. *P<0.05, **P<0.01, ***P<0.001; NS, non significant by two-tailed *t* test.

When seeded onto fibroblasts, endothelial cells initially form clusters which remodel and sprout to form a network of tubules^15^; lumenized tubes form following suppression of sprouting, driven by establishment of mature endothelial cell adherens junctions^4^ (Supplementary Fig. 1a–d). Knockdown of DOCK4 by RNAi reduced the number of clusters detected at 3 days after seeding human umbilical vein endothelial cells (HUVEC) onto fibroblasts (Fig. 1a and Supplementary Fig. 1f). Early association of endothelial cells, junctional organization of VE-cadherin and VE-cadherin expression levels were unaffected by DOCK4 knockdown (Fig. 1b and Supplementary Fig. 1g), suggesting that DOCK4 is not required for VE-cadherin function *per se*; DOCK4 knockdown did not affect endothelial cell proliferation at that stage (Supplementary Fig. 1h) or the spreading and bipolarity of single endothelial cells (Supplementary Fig. 1i). However tubules developed at a faster rate, with endothelial cells within tubules appearing less spread and more polarized at earlier timepoints (Fig. 1b). These observations show that DOCK4 regulates the shape of endothelial cells in the developing tubules but is dispensable for initial stages of tubule morphogenesis while subsequent steps are perturbed with knockdown of DOCK4: sprouting was blocked (Fig. 1c); the developed tubules were thinner with fewer lateral cell–cell contacts (Fig. 1d). There was 40% decrease in lumenized tubes; lumenless cords lacked apposing endothelial cells and apical organization of podocalyxin (Fig. 1e and Supplementary Movie 2).

To gain insight into the behaviour of tubules formed in the absence of DOCK4 and loss of lateral contacts, we followed HUVEC in coculture by time-lapse microscopy. Control tubules remodelled actively and sprouted through dynamic protrusions while maintaining thickness (Fig. 1f and Supplementary Movies 3 and 5). Tubules with DOCK4 knockdown did not remodel or sprout but elongated in a unidirectional manner and became thinner with time (Fig. 1f and Supplementary Movies 4 and 6). Protrusive activity was blocked (Fig. 1f); initiation of protrusions was observed occasionally in the absence of DOCK4; however, those were not productive and failed to remodel the tubules (Fig. 1f and Supplementary Movie 4); anastomosis proceeded in the absence of DOCK4 (Fig. 1g, Supplementary Fig. 1j and Supplementary Movies 5 and 6). Altogether, the data show that DOCK4 is necessary for endothelial cell protrusive activity and tubule remodelling, organization of lateral contacts and lumen formation, but is dispensable for tubule elongation and anastomosis.

### RhoG and Cdc42 are required for tubule formation

**Figure 2:**
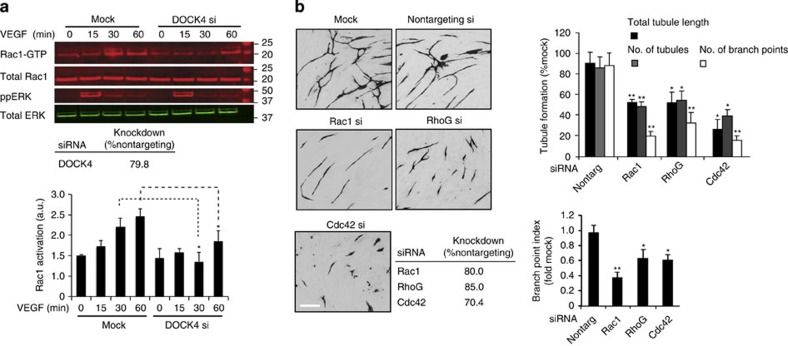
Rac activation controls tubule formation with RhoG and Cdc42 (**a**) Rac1 activation (GTP-bound Rac/total Rac) following DOCK4 depletion (si, smartpool siRNA) in HUVEC stimulated with VEGF (25 ng ml^−1^), bars indicate s.e.m.; *n*=4 independent experiments. Rac1 activation following shRNA-mediated DOCK4 depletion is shown in Supplementary Fig. 1k. (**b**) Images of organotypic cocultures (CD31 staining) show tubule morphology following Rho GTPase depletion (siRNA, si) in HUVEC 5 days after seeding onto CFs. Scale bar, 200 μm. Top histogram: quantification of total tubule length, number of tubules, number of branch points±s.e.m.; *n*=number of independent experiments (nontargeting, *n=*4; Rac1, *n=*3; RhoG, *n=*4; Cdc42, *n=*3); three cocultures quantified in each experiment. Table shows knockdown quantified by quantitative PCR. Lower histogram: branch point index. Bars represent junctions divided by tubule length±s.e.m.; *n*=number of independent experiments as in **b**. *P<0.05; **P<0.01 by two-tailed *t*-test compared with nontargeting control.

DOCK4 is a member of the DOCK-B subfamily of Rac GEFs^16^. We studied whether DOCK4 acts as a Rac1 GEF downstream of the major angiogenic growth factor VEGF. Rac1 activation assays showed knockdown of DOCK4 blocks VEGF-mediated Rac1 activation (Fig. 2a and Supplementary Fig. 1k). Consistent with DOCK4 acting as a Rac1 GEF, Rac1 but not RhoA or Cdc42 interacted with DOCK4 under conditions that stabilize the GTPase nucleotide-free state and interaction with GEFs (Supplementary Fig. 1l). Knockdown of Rac1 resulted in distinct linear tubule morphology with pronounced reduction in the number of branches (Fig. 2b and Supplementary Fig. 2a). Supplementary Figure 2b shows by quantification from time-lapse movies (Supplementary Movies 7 and 8) blockade of protrusions on tubules with knockdown of Rac1. This defect in protrusive activity with Rac1 knockdown, like knockdown of DOCK4, was not associated with defective migratory behaviour of single endothelial cells in the organotypic coculture (Supplementary Fig. 2c). To investigate which other Rho-GTPases are involved in tubule formation, we performed RNAi screens. Knockdown of a number of Rho-GTPases perturbed tubule formation to different extents (Supplementary Table 2). Knockdown of RhoG reduced the overall levels of tubule formation to a similar extent as Rac1 (Fig. 2b and Supplementary Fig. 2d) although individual tubules appeared shorter (Fig. 2b). There was marked blockade of tubule formation in response to Cdc42 knockdown (Fig. 2b and Supplementary Fig. 2e) associated with inhibition of spreading and migration of single endothelial cells (Supplementary Fig. 2f,g). These data show that Rac1, RhoG and Cdc42 are key regulators of tubule formation and that they control diverse endothelial cell processes.

### DOCK4 controls lateral filopodia formation

**Figure 3:**
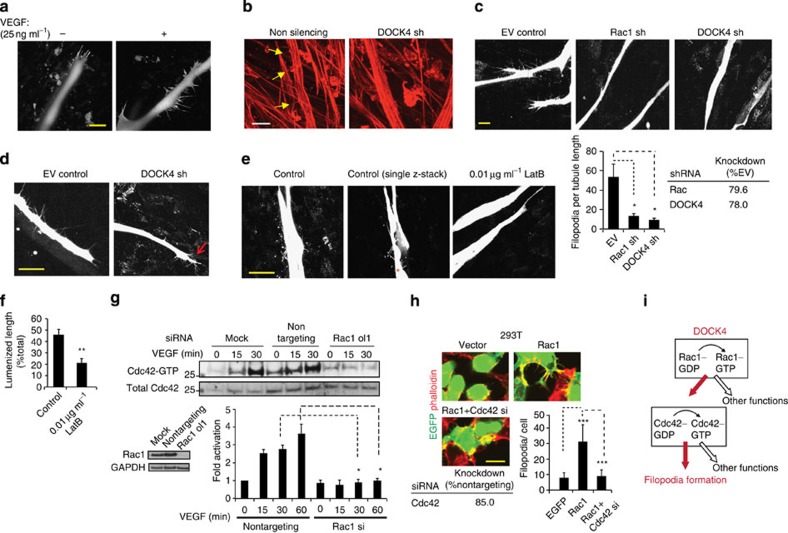
DOCK4 is required for lateral filopodia formation and Cdc42 activation (**a**) Confocal images of filopodia in organotypic cocultures following VEGF stimulation (25 ng ml^−1^). HUVEC–EGFP were seeded onto CFs and after 4 days the culture media were replenished with VEGF-containing media. Cocultures were imaged after 24 h. Scale bar, 50 μM. (**b**) Confocal images of phalloidin staining of filopodia (yellow arrows) in the presence of VEGF (25 ng ml^−1^) 6 days after seeding HUVEC with DOCK4 depletion (EV, empty vector; sh, shRNA1) onto CFs. Scale bar, 25 μM. (**c**) Confocal images of filopodia as in **b** after depletion of Rac1 or DOCK4 (EV, empty vector; sh1, shRNA1). Images are maximum intensity projections of confocal Z-stacks. Scale bar, 50 μM. Histogram: filopodia quantifications, error bars indicate s.e.m.; *n*=number of organotypic cocultures (EV, *n=*8; Rac sh1, *n=*6; DOCK4 sh1, *n=*6) from independent experiments (EV, *n=*3; Rac sh1, *n=*2; DOCK4 sh1, *n=*3). Knockdown was quantified by immunoblot (Supplementary Figs 1k and 2a). (**d**) Confocal images show persisting tip filopodia (red arrow) in tubules with DOCK4 depletion as in **b**. Scale bar, 50 μM. (**e**) Treatment with 0.01 μg ml^−1^ Latrunculin B (LatB) blocks lateral filopodia. Cocultures were treated for 48 h starting at 6 days after seeding HUVEC onto CFs. Middle panel shows filopodia in single confocal Z-stack (asterisk, focus level). Scale bar, 50 μM. (**f**) Histogram: quantification of lumenized length 14 days after seeding HUVEC onto CFs and following 48 h 0.01 μg ml^−1^ LatB treatment at 3, 6 and 9 days (6 days total treatment) compared with untreated control. Each value represents the lumenized length as percentage of total length±s.e.m.; *n*=4 organotypic cocultures for each condition. Supplementary Figure 3c,d shows adherens junctions and thin tubule morphology with LatB treatment. **(g)** Immunoblots of Cdc42 activation with VEGF stimulation (25 ng ml^−1^) in HUVEC after depletion of Rac1 (ol1, siRNA oligonucleotide 1). Histogram: fold Cdc42 activation (GTP-bound Cdc42/total Cdc42) following Rac1 depletion (si, siRNA oligonucleotide 1), error bars indicate s.e.m.; *n*=3 independent experiments; Supplementary Fig. 3e shows Cdc42 activation with shRNA-mediated Rac1 knockdown. **(h**) Images of phalloidin-stained 293T cells following overexpression of EGFP–Rac1 and knockdown of Cdc42 (si, siRNA). Vector (EGFP) and EGFP–Rac1 (top panels) were co-transfected with nontargeting siRNA. Images of wider areas in Supplementary Fig. 3g. Scale bar, 25 μM. Histogram: filopodia quantifications, error bars indicate s.e.m.; *n*=30 cells from two independent experiments. (**i**) DOCK4→Rac1→Cdc42 signalling module regulates filopodia formation. *P<0.05, **P<0.01, ***P<0.001 by two-tailed *t* test.

Knockdown of DOCK4 resulted in loss of dynamic remodelling and protrusive activity. Protrusions and spouts in the coculture system, like *in vivo* are led by filopodia^5^,^7^ (Fig. 3a–c). At early stages of tubule formation, VEGF promotes lateral filopodia for generation of sprouts (Fig. 3a). Filopodia in the coculture system are actin rich as shown by phalloidin staining (Fig. 3b) and sites of localization of VE-cadherin (Supplementary Fig. 3a); filopodia extend and retract dynamically at the tips of developing tubules and at lateral sites (Supplementary Movie 9). Strikingly, knockdown of DOCK4 blocked filopodia and sprouts (Fig. 3b,c), as did knockdown of Rac1 (Fig. 3c). While DOCK4 knockdown abolished lateral filopodia (Fig. 3b,c), filopodia persisted at the tips of tubules (Fig. 3d and Supplementary Movie 10), suggesting distinct mechanisms of control at tip and lateral sites. Bleb-like membrane protrusions were observed on some tubules with DOCK4 knockdown (Supplementary Movie 10) potentially indicative of increased actomyosin contractility retracting filopodia; however, treatment of the cocultures with the ROCK inhibitor Y27632, which blocks actomyosin contractility^4^, did not reverse the blockade of filopodia (Supplementary Fig. 3b). Altogether, these observations suggest that (i) the defect in tubule remodelling with knockdown of DOCK4 is due to loss of filopodia and protrusions, (ii) DOCK4 controls filopodia via a direct mechanism.

Filopodia formation and protrusive activity require rearrangements of the actin cytoskeleton^23^. To investigate the role of actin remodelling in these processes, we treated the cocultures with low concentrations of the inhibitor of actin polymerization Latrunculin B (LatB)^24^ to block filopodia without affecting endothelial cell viability and adherens junction formation^7^. LatB at 0.01 μg ml^−1^ blocks lateral filopodia (Fig. 3e). Treatment of the cocultures with LatB blocked sprouts, the tubules were long and thin with fewer lateral contacts resembling tubules with DOCK4 knockdown (Supplementary Fig. 3c,d). The treatment attenuated lumen formation (Fig. 3f). These experiments show that the dynamic remodelling and organization of endothelial cells in developing tubules requires protrusive activity led by filopodia, and that blockade of cytoskeletal remodelling and protrusive activity early in tubule morphogenesis influences subsequent stages of lumen formation.

It is well established that the formation of filopodia depends on Cdc42 (ref. 25). Therefore, we investigated whether Rac1 controls Cdc42 in endothelial cells. VEGF stimulation strongly activated Cdc42; strikingly, knockdown of Rac1 abolished Cdc42 activation (Fig. 3g and Supplementary Fig. 3e), while knockdown of Cdc42 had little effect on Rac1 (Supplementary Fig. 3f). These data suggest that Cdc42 activation for filopodia formation depends on Rac1. To corroborate this finding in another cell system, we performed experiments in a non-endothelial cell type: 293T cells. Those experiments showed that overexpression of Rac1 stimulated filopodia formation that was blocked by knockdown of Cdc42 (Fig. 3h and Supplementary Fig. 3g), confirming interplay between Rac1 and Cdc42 activation in filopodia formation (Fig. 3i).

### RhoG controls Rac1 activation and filopodia formation

**Figure 4:**
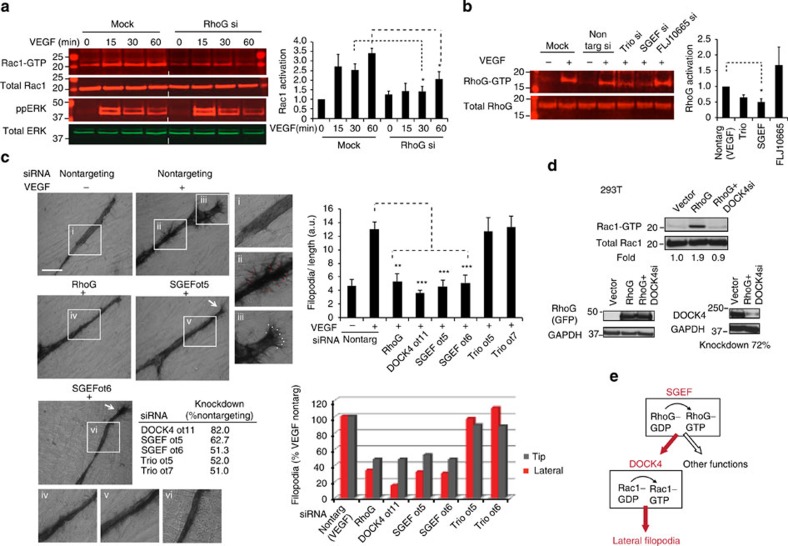
RhoG and SGEF control Rac1 activation and lateral filopodia formation (**a**) Immunoblots of Rac1 activation on VEGF stimulation (25 ng ml^−1^) in HUVEC following RhoG depletion (si, siRNA smartpool). Histogram: fold increase of Rac1 activation (GTP-bound Rac/total Rac) on VEGF stimulation. Error bars represent s.e.m.; *n*=independent experiments (15 min, *n*=3; 30 min and 60 min, *n*=5). Supplementary Figure 4a shows RhoG requirement for Rac activation following depletion with on-target oligonucleotides. (**b**) Immunoblots show levels of activated RhoG in HUVEC after 30 min VEGF stimulation (25 ng ml^−1^) and following depletion of Trio, SGEF or FLJ10665 (si, siRNA smartpool). Histogram: fold RhoG activation (GTP-bound RhoG/total RhoG) compared with VEGF-treated nontargeting control. Error bars represent s.e.m. (*n*=3 independent experiments). Supplementary Figure 4c shows blockade of VEGF-stimulated RhoG activation on SGEF depletion with on-target oligonucleotides. (**c**) Images show loss of filopodia by CD31 staining following RhoG or SGEF depletion (ot, on-target plus oligonucleotides; si, siRNA smartpool) in cocultures treated with VEGF (Supplementary Fig. 1a) 5 days after seeding HUVEC onto CFs. Arrows: tip filopodia; (i–iv): magnifications of outlined areas in main images. Scale bar, 100 μm. Histograms: upper panel, quantification of total filopodia; lower panel, quantification of lateral filopodia (red dots) and tip filopodia (white dots). Error bars s.e.m.; *n*=number of organotypic cocultures (Scr±VEGF, *n=*7; RhoG, *n=*5; SGEFot5, *n=*6; SGEFot6, *n=*6; DOCK4ot11, *n=*3; Trio ot5, *n=*6; Trio ot7, *n=*6) from independent experiments (Scr±VEGF, *n=*3; RhoG, *n=*2; SGEFot5, *n=*3; SGEFot6, *n=*3; DOCK4ot11, *n=*2; Trio ot5, *n=*3; Trio ot7, *n=*3). Table shows knockdown quantified by quantitative PCR. (**d**) Immunoblots in upper panels: Rac1 activation in HEK293T cells with RhoG overexpression; lower panels: RhoG and DOCK4 levels by western blot. (**e**) RhoG→DOCK4→Rac1 signalling module controls lateral filopodia formation. *P<0.05, **P<0.01, ***P<0.001 by two-tailed *t*-test compared with indicated controls.

Rac1 activation through DOCK180 family GEFs requires RhoG^26^,^27^. We therefore investigated whether RhoG controls DOCK4-dependent Rac1 activation and filopodia formation in endothelial cells. RNAi-mediated knockdown of RhoG blocked VEGF-driven Rac1 activation (Fig. 4a and Supplementary Fig. 4a). We established that VEGF activates RhoG (Supplementary Fig. 4b) and investigated which RhoG GEFs control RhoG activation downstream of VEGF signalling. RNAi-mediated knockdown of SGEF but not FLJ1066 and to a lesser extent Trio reduced activated RhoG levels (Fig. 4b and Supplementary Fig. 4c). Knockdown of RhoG resulted in reduction in branches and overall tubule length (Fig. 2b); in tubules that retained length there was blockade of lateral filopodia (Fig. 4c). Knockdown of Trio did not affect filopodia or branches (Fig. 4c and Supplementary Fig. 4d), suggesting it may control a different RhoG-dependent process. Knockdown of SGEF, as with the knockdown of DOCK4, blocked lateral filopodia and to a lesser extent tip filopodia (Fig. 4c); tubules with SGEF knockdown showed more linear tubule morphology compared with controls and decrease in branches (Supplementary Fig. 4d). These data suggest that SGEF and RhoG operate upstream of Rac1 and DOCK4 to control lateral filopodia and branches. To confirm that RhoG, DOCK4 and Rac1 operate in the same signalling module, we performed overexpression experiments in 293T cells. Overexpression of RhoG resulted in increased Rac1 activation that was blocked by knockdown of DOCK4 (Fig. 4d). Altogether the data argue for a RhoG→DOCK4→Rac1 signalling module downstream of VEGF and in 293T cells (Fig. 4e).

### Rac GEF DOCK4 is in a complex with Cdc42 GEF DOCK9

**Figure 5:**
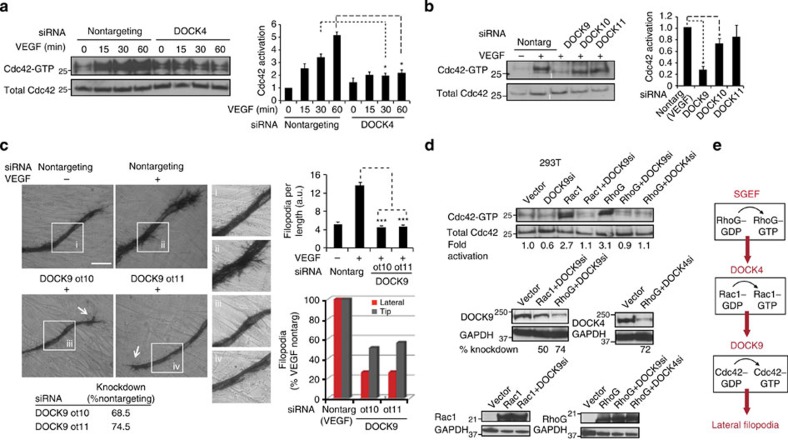
DOCK4 is required for DOCK9-dependent activation of Cdc42 and filopodia formation (**a**) Immunoblots of Cdc42 activation on VEGF stimulation (25 ng ml^−1^) in HUVEC following DOCK4 depletion (smartpool siRNA). Histogram: fold Cdc42 activation (GTP-bound Cdc42/total Cdc42) in HUVEC after DOCK4 depletion; error bars indicate s.e.m.; *n*=3 independent experiments). Supplementary Figure 5a shows blockade of Cdc42 activation on DOCK4 depletion with an on-target oligonucleotide. (**b**) Immunoblots show activated Cdc42 levels in HUVEC after 30 min VEGF stimulation (25 ng ml^−1^) and following depletion of DOCK9, DOCK10 or DOCK11 (si, siRNA smartpool). Histogram: fold Cdc42 activation (GTP-bound Cdc42/total Cdc42) compared with VEGF-treated nontargeting control. Error bars s.e.m. (*n*=3 independent experiments). (**c**) Images of tubules (CD31 staining) show filopodia formation following DOCK9 depletion (ot, on-target plus oligonucleotides; si, siRNA smartpool) in cocultures treated with VEGF (Supplementary Fig. 1a) 5 days after seeding onto CFs visualized by CD31 staining. Arrows: tip filopodia; (i–vi): magnifications of boxes in main images. Scale bar, 100 μm. Histograms: quantification of total filopodia (upper panel) and lateral or tip filopodia (lower panel). Error bars represent s.e.m.; *n*=number of organotypic cocultures (Scr±VEGF, *n=*6; DOCK9ot10, *n=*6; DOCK9 ot11, *n=*5) from independent experiments (Scr±VEGF, *n=*3; DOCK9ot10, *n=*3; DOCK9 ot11, *n=*2). Knockdown was quantified by quantitative PCR. (**d**) Immunoblots in upper panels: Cdc42 activation in 293T cells with Rac1 and RhoG overexpression; immunoblots in middle and lower panels: levels of Rac1, RhoG, DOCK4 and DOCK9. (**e**) SGEF→RhoG→DOCK4→Rac1→DOCK9→Cdc42 signalling module controls lateral filopodia formation. *P<0.05, **P<0.01, ***P<0.001 by two-tailed *t*-test compared with indicated controls.

We sought to gain understanding of the molecular mechanism of Cdc42 and Rac interplay and how it influences filopodia and tubule formation. First we showed that knockdown of DOCK4 in endothelial cells blocks Cdc42 activation downstream of VEGF (Fig. 5a and Supplementary Fig. 5a). We then investigated which GEF controls Cdc42 activation. We screened a number of GEFs including DOCK9, DOCK10, DOCK11, FGD3 and FGD6, on the rationale that either they are Cdc42 GEFs or because their knockdown resulted in tubule morphology similar to knockdown of Cdc42 in the RNAi screen (Supplementary Table 1). Knockdown of DOCK9 blocked VEGF-driven Cdc42 activation (Fig. 5b and Supplementary Fig. 5b). Knockdown of DOCK9 inhibited lateral filopodia and to a lesser extent tip filopodia (Fig. 5c); tubules with DOCK9 knockdown showed more linear tubule morphology compared with controls (Supplementary Fig. 5c). In 293T cells, Rac1 overexpression increased Cdc42 activation that was blocked by knockdown of DOCK9 (Fig. 5d). Consistent with RhoG acting upstream of DOCK4 and Rac1, RhoG overexpression resulted in increased Cdc42 activation that was blocked by knockdown of DOCK4 or DOCK9 (Fig. 5d). Altogether, the data delineate a signalling module, SGEF→RhoG→DOCK4→Rac1→DOCK9→Cdc42 downstream of VEGF, which controls lateral filopodia formation (Fig. 5e).

**Figure 6:**
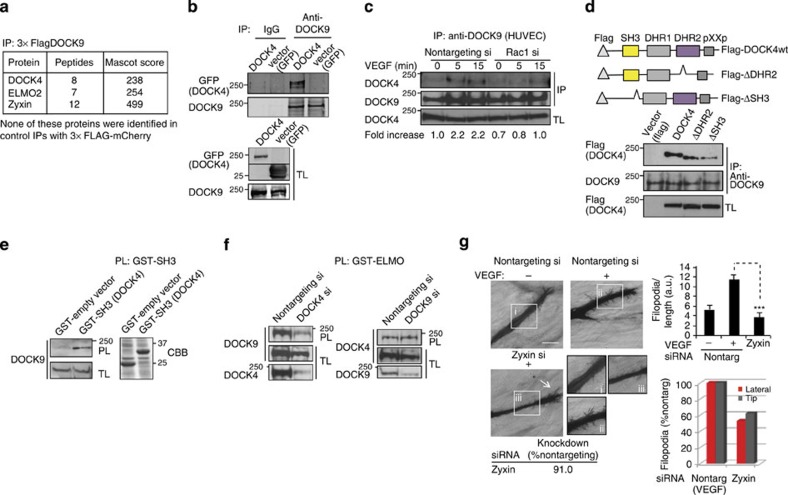
Rac GEF DOCK4 and Cdc42 GEF DOCK9 are in a complex (**a**) Mass spectrometry analysis following IP from 293T cells of overexpressed 3 × Flag–DOCK9 (DOCK9 interaction partners in Supplementary Table 3). (**b**) Upper panels show immunoblot of DOCK4 in DOCK9 or control IgG immunoprecipitated complexes (IPs) following GFP–DOCK4 and empty vector (GFP) overexpression and immunoprecipitated DOCK9 in the IPs. Lower panels show overexpressed GFP–DOCK4 and empty vector (GFP) in total lysate (TL). (**c**) Immunoblot of DOCK4 in DOCK9 IPs following VEGF stimulation (25 ng ml^−1^) and after Rac1 knockdown (Rac1 si, siRNA ol1). Fold increase of DOCK4–DOCK9 interaction compared with unstimulated control after normalization for DOCK9 in IP and DOCK4 in total lysate (TL). (**d**) Immunoblot of Flag-DOCK4 in DOCK9 IPs following overexpression of DOCK4 wildtype or deletion mutants in 293T shows the DOCK4-SH3 and DHR2 domains are required for interaction with DOCK9. (**e**) Immunoblot of DOCK9 in GST–SH3 pulldowns (PLs). Right hand panel: GST proteins visualized by Coomasie Brilliant Blue (CBB) staining. (**f**) Immunobots of DOCK9 (left panels) and DOCK4 (right panels) in GST–ELMO PLs following knockdown of DOCK4 and DOCK9, respectively, show DOCK4 is necessary for interaction of DOCK9 with ELMO. (**g**) Filopodia formation following zyxin depletion (si, siRNA smartpool) in cocultures treated with VEGF (Supplementary Fig. 1a) 5 days after seeding onto CFs visualized by CD31 staining. Arrow: tip filopodia. Scale bar, 100 μm. Histograms: quantification of total filopodia (upper panel) and lateral or tip filopodia (lower panel). Error bars s.e.m.; *n*=4 organotypic cocultures from two independent experiments. Knockdown was quantified by quantitative PCR. *P*<0.001 by two-tailed *t*-test compared to the indicated control.

To gain insight into how Rac1 activation might lead to activation of Cdc42, we isolated interaction partners of DOCK9 by pull-down assay in 293T cells and analysed the complexes by liquid chromatography tandem mass spectrometry (LC-MS/MS). Analysis showed there is interaction between DOCK4 and DOCK9 (Fig. 6a) that was corroborated by western blotting: DOCK4 was detected in DOCK9-immunoprecipitated complexes following overexpression of DOCK4 (Fig. 6b); association of endogenous DOCK4 with endogenous DOCK9 was detected in HUVEC in the presence of VEGF that was blocked with knockdown of Rac1 (Fig. 6c). We investigated further the interaction between DOCK4 and DOCK9. Members of the DOCK-A and DOCK-B subfamilies including DOCK4 can homodimerize via their DHR2 domains^28^ and may interact with ELMO via their Src homology 3 (SH3) domains^26^,^29^,^30^. Interaction between DOCK4 and DOCK9 required both the DOCK4 DHR2 and SH3 domains (Fig. 6d) with the minimal SH3 domain sufficient for association (Fig. 6e). These data show that in addition to its involvement in the interaction with ELMO, the DOCK4-SH3 domain also mediates interaction with DOCK9. This is in agreement with the MS analysis, which identified ELMO as an interaction partner of DOCK9 (Fig. 6a). DOCK9 lacks an SH3 domain, suggesting its association with ELMO is indirect. Pull-down assays using GST–ELMO, showed DOCK9 interaction with ELMO is through DOCK4 since knockdown of DOCK4 reduced binding of DOCK9 to ELMO, while the DOCK4–ELMO interaction (Supplementary Fig. 5d) was unaffected by knockdown of DOCK9 (Fig. 6f). The MS analysis identified a number of other DOCK9-interacting proteins that included zyxin (Supplementary Table 3 and Fig. 6a), a component of filopodial focal complexes and regulator of actin polymerization^31^,^32^. Knockdown of zyxin attenuated filopodia formation (Fig. 6g). Altogether, the interaction data argue for DOCK4 homodimers interacting with ELMO and DOCK9 via the DOCK4-SH3 domains and DOCK9 interacting with zyxin to directly influence actin dynamics.

### DOCK4 controls blood vessel lumen formation *in vivo*

**Figure 7:**
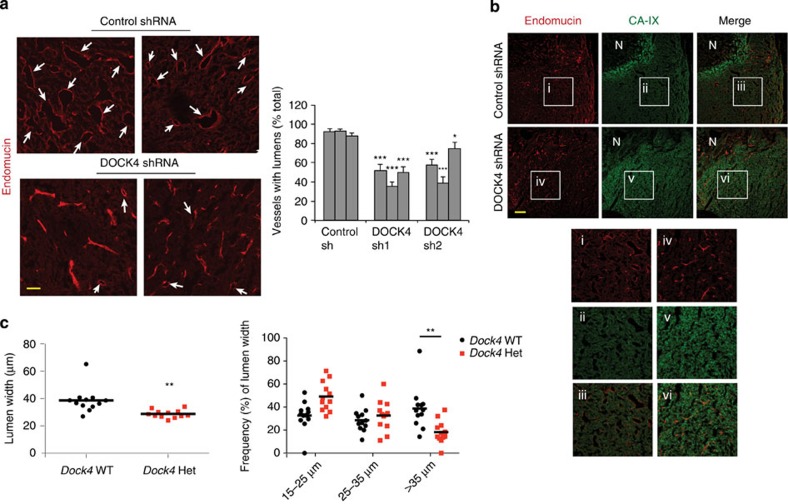
DOCK4 controls lumen formation in tumours (**a**) Images of sections immunostained with endomucin to visualize blood vessels in BE xenograft tumours following DOCK4 shRNA transduction of the vascular compartment. Scale bar, 50 μm. Histogram: counts of lumenized vessels as %total vessel count in individual tumours, error bars indicate s.e.m.; *n*=12 microscopic fields representing three different tumour levels. (**b**) CAIX staining of tumour sections to show hypoxic regions. Scale bar, 200 μm. (**c**) Lumen analysis in EO771 tumours implanted in *Dock4* wildtype (WT) and heterozygous (Het) mice (three tumours from each condition). Left scatter plot: average lumen width; right scatter plot: frequency of lumen size; *n*=4 tiled images for each tumour representing two tumour levels, each image across a tumour section. *P<0.05, **P<0.01, ***P<0.001 by two-tailed *t*-test compared with indicated controls.

The *in vitro* analyses suggested an *in vivo* role for DOCK4 in new vessel formation. DOCK4 expression was abundant in tumour blood vessels *in vivo* (Supplementary Fig. 6); therefore, we investigated if DOCK4 controls lumen formation in tumours. First we used a xenograft tumour model where s.c. injection of tumour cells with irradiated ecotropic retrovirus producers results in transduction of the host vasculature and endothelial-specific gene expression^15^. We used this approach to deliver short hairpin RNAs (shRNAs) for knockdown of DOCK4 in the vasculature of BE colorectal tumours^15^. Delivery of two different DOCK4 shRNAs using this approach resulted in changes in the morphology of tumour blood vessels (Fig. 7a) consistent with performance of the co-injected retroviral producer lines (Supplementary Fig. 7a). Tumours DOCK4 shRNAs targeted to the vasculature lacked large calibre lumens compared with controls (Fig. 7a). To evaluate vessel functionality, we assessed hypoxia by staining tumour sections for carbonic anhydrase CA-IX^33^. Control tumours had CA-IX-positive hypoxic regions around the necrotic centre (Fig. 7b); necrotic regions were less prominent with DOCK4 shRNA, although hypoxia was observed in areas lacking larger lumens (Fig. 7b). The data show that DOCK4 is required for generation of blood vessel lumens in tumours and that changes in blood vessel calibre by knockdown of DOCK4 impact on tumour hypoxia.

To confirm these results by means of genetic deletion, we generated a *Dock4* constitutive mouse knockout line (Supplementary Fig. 7b,c). Homozygous deletion of *Dock4* leads to early embryonic lethality (Supplementary Fig. 7d); therefore, we employed *Dock4* heterozygous mice in tumour experiments. Heterozygous mice and wild-type controls were implanted intracranially with a syngeneic breast cancer cell line (EO771 flucII) which gives rise to highly vascularized tumours in the brain (M. Lorger, unpublished data). Vessel morphology varied in the tumours, with areas of lumenized large calibre vessels and areas of small capillaries (Supplementary Fig. 8a,b). Analysis of all identifiable lumenized vessels in tumour sections showed decrease in lumen size in tumours grown in *Dock4* heterozygous mice compared with controls (Fig. 7c, and Supplementary Fig. 8b). In control tumours, ∼38% of vessels with lumens had larger calibre (>35 μm), compared with 21% in tumours grown in heterozygous *Dock4* null mice (Fig. 7c), corroborating findings in the xenograft model. In developmental angiogenesis, a similar effect in blood vessel lumen size was detected in the brain parenchyma of E13.5 *Dock*4 heterozygous embryos (Supplementary Fig. 9a). In controls, ∼31% of vessels with lumens had relatively large calibre (>20 μm), whereas in heterozygous *Dock*4 null mice the percentage of vessels with larger lumens was 9%. Differences in lumen size were not associated with changes in pericyte coverage in wild-type or *Dock4* heterozygous embryos (Supplementary Fig. 9b). Differences were not detected in the calibre of the aortas of heterozygous *Dock*4 null mice (Supplementary Fig. 9c), potentially indicating that different molecules operate in the aorta and the brain microvasculature during blood vessel lumen formation. Although we cannot exclude contribution of non-vascular cell types in the observed effects of DOCK4 genetic deletion in blood vessel development, altogether the data show that DOCK4 regulates blood vessel lumens in tumours and during development in *vivo*.

## Discussion

**Figure 8:**
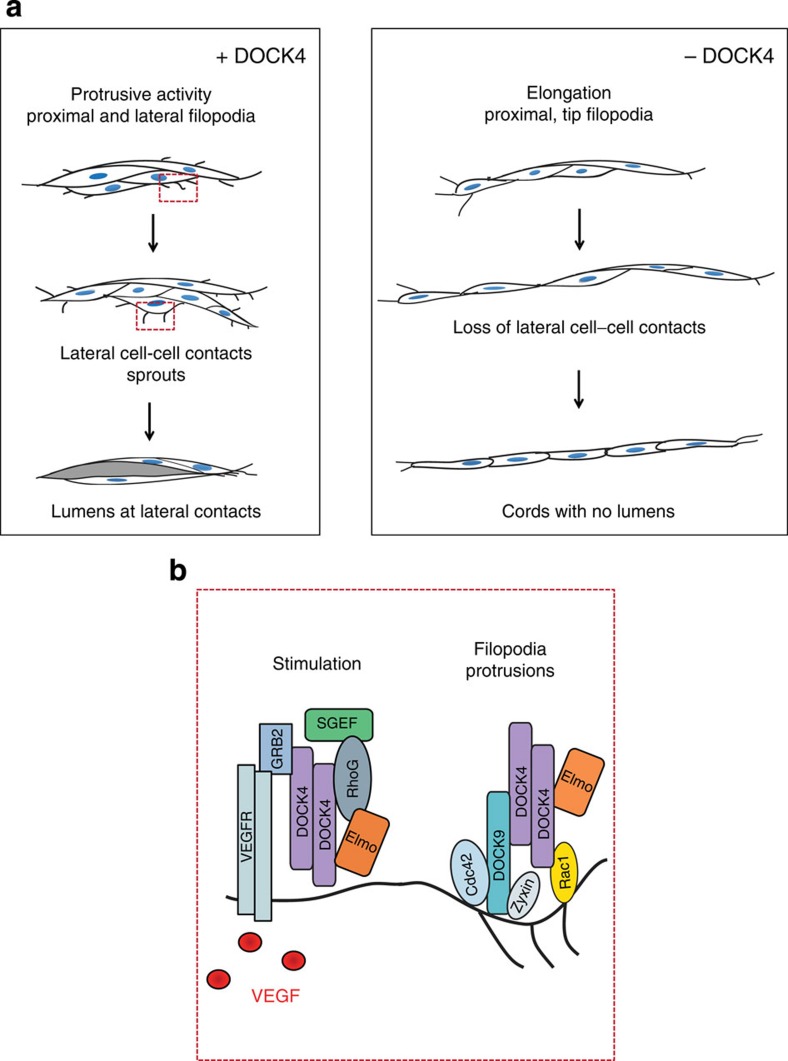
Model of regulation of filopodia formation and lumen morphogenesis by Rac GEF DOCK4 (**a**) Stages of tubule development and lumen formation in the presence or absence of DOCK4. Dynamic remodelling via lateral filopodia and protrusions leads to formation of lumens lined by apposing endothelial cells. In the absence of DOCK4, lack of dynamic protrusive activity and dynamic remodelling leads to thin tubules lacking lateral cell–cell contacts which do not form a lumen. (**b**) Signalling downstream of VEGF activates the SGEF→RhoG→DOCK4→Rac1→DOCK9→Cdc42 signalling pathway and promotes interaction of Rac GEF DOCK4 with Cdc42 DOCK9.

We show that DOCK4 controls tubule filopodia and protrusions required for the dynamic behaviour of endothelial cells in developing tubules and for lumen formation (Fig. 8a). This finding is unexpected: DOCK4 is a member of a family of Rac GEFs^16^,^34^ which have previously been implicated in the control of lamellipodia formation^19^,^27^, rather than filopodia formation, which is driven by Cdc42 (ref. 35). However, studies in *Drosophila* have shown that during dorsal closure, Rac1 is required for formation of filopodial structures and that expression of constitutively active Rac induces prominent filopodia alongside large lamellipodia^36^, thereby implicating Rac1 in the control of both lamellipodia and filopodia. Studies in mammalian cells employing dominant negative mutants, and genetic studies in *Caenorhabditis elegans* suggest that Rac operates downstream of Cdc42 (refs 25, 37). However, we show that Rac1 activation by DOCK4 is required for Cdc42 activation by the GEF DOCK9. Importantly, we demonstrate that DOCK4 is in a complex with DOCK9, an association dependent on the amino terminal SH3 domain of DOCK4. The SH3 domain of DOCK family GEFs is known to interact with sequences in ELMO proteins containing proline-rich (pXXp) motifs^29^; several such pXXp motifs are present in the protein sequence of DOCK9 that may mediate the interaction with DOCK4. As the DOCK4 DHR2 domain is also necessary for the DOCK4–DOCK9 interaction, we propose that homodimerization of DOCK4 mediated by the DHR2 domain^38^ permits one DOCK4-SH3 domain in the homodimer to interact with ELMO and one with DOCK9 (Fig. 8b). This is supported by the association between DOCK9 and the DOCK4-SH3 domain (Fig. 6e), and the association between DOCK9 and ELMO being dependent on DOCK4 (Fig. 6f). These findings present a paradigm of how activity of GTPases with apparent distinct functions can be co-ordinately regulated via association of their respective GEFs to control common cellular processes.

We demonstrate that VEGF activates a RhoG→DOCK4→Rac1→DOCK9→Cdc42 signalling module that controls lateral filopodia formation, while some tip filopodia persist when this signalling pathway is blocked. These filopodia may present a subpopulation with distinct functions, as reported previously in other cell systems^39^, for example, they may promote spreading and survival of tip cells. The data suggest that alternative molecular mechanisms are in play for those tip filopodia which may depend on PI3K signalling and the downstream effector ARAP3 (ref. 40, 41). Alternatively, tip filopodia may require different thresholds of VEGF and/or different rates of actin polymerization and stability, or may be regulated by different extracellular cues. Activation of the signalling pathway may be through DOCK4 association with VEGFR2, either directly or through an adaptor such as Grb2 which is known to interact with DOCK pXXp motifs^42^. Interaction would then promote association of DOCK4 with DOCK9 and ELMO for translocation of the complex to the plasma membrane following RhoG activation^27^. Our studies show that RhoG and the RhoG GEF, SGEF, regulate the DOCK4→Rac1→DOCK9→Cdc42 signalling module. *RhoG* and *SGEF* knockout mice are viable^43^,^44^, suggesting that vascular defects are mild or selective pressure for generation of a functional vasculature can overcome requirement for upstream regulators *in vivo*. Although knockdown of SGEF, DOCK4 or DOCK9 in endothelial cells in the coculture system had similar effects on filopodia formation and tubule morphology, the GTPases play additional roles: Cdc42 is necessary for endothelial cell spreading and migration, whereas RhoG may also control tubule length. These observations support the notion that GTPases integrate signals from multiple pathways while the GEFs act to transduce specific signals. Rho proteins may act in a signalling pathway to orchestrate and to fine-tune signal transduction by providing multiple points of integration, and to coordinate the action of multiple effectors for control of complex cellular processes.

Knockdown of DOCK4 resulted in loss of lateral contacts and inhibition of lumen formation*. Dock*4 homozygous genetic deletion in mice is embryonic lethal prior to the onset of vascularization, precluding analysis of vascular phenotypes. Both global genetic deletion of a single *Dock4* allele in tumour-bearing mice and targeted knockdown in the vasculature of xenograft tumours perturbed blood vessel lumen formation, supporting the findings in the tissue culture angiogenesis model. Vascular lumens *in vivo* arise through different mechanisms^45^: extension of a transcellular lumen through coalescing intracellular vacuoles^46^,^47^; or intercellular hollowing, through separation of apposing endothelial cells and redistribution of pre-formed junctions to the periphery of the developing tube^21^,^48^. The latter requires polarization in the apical-basal axis and lateral cell–cell contacts^21^,^48^, a feature of sprouting tubules in tissue culture and angiogenic blood vessels *in vivo* including intersegmental vessels in zebrafish embryos and retinal vessels in mice^48^,^49^. Knockdown of DOCK4 in the coculture assay resulted in a loss of dynamic tubule remodelling, affecting lateral cell–cell contacts, followed by defective lumen formation. The unidirectional tubule elongation associated with this loss of lateral filopodia and protrusive activity following knockdown of DOCK4, resembles the directionally persistent cell motility described previously following Rac1 inhibition^50^. This suggests that Rac activity must be precisely regulated to balance tubule elongation and lateral protrusions. Our study shows that filopodia-led protrusions and cytoskeletal remodelling are necessary for the correct organization of endothelial cells in the developing tubules, and that blocking these processes early in tubule formation impacts on lumen morphogenesis.

## Methods

### Cell culture and antibodies

Human dermal fibroblasts (HDF) and HUVEC were from TCS CellWorks (Buckingham, UK) and were cultured in DMEM containing 10% FCS and Human Large Vessel Endothelial Cell Growth Medium (TCS CellWorks). HUVEC–enhanced green fluorescent protein (EGFP)^15^ was generated by infection with a retrovirus harbouring EGFP followed by fluorescence-activated cell sorting. HUVEC were used to passage 6 and HDF between passages 7 and 11. HEK293T cells were purchased from Clontech Laboratories, cultured in DMEM containing 10% FCS, expanded on collagen-coated plates for the first two passages according to the Clontech protocol and used to passage 8. NIH 3T3 fibroblasts were from C.J.M. and were cultured in DMEM containing 10% FBS; BE colon carcinoma cells, originally from the American Type Tissue Collection, were from C.J.M. and were cultured in DMEM containing 10% FCS. EO771 flucII cells (C57BL/6J breast cancer cell line) were obtained from M.L. and were cultured in RPMI 20% FBS, 1 mM sodium pyruvate, 2 mM L-Glutamine and 1 × non-essential amino acids.

Antibodies used in these studies were sourced as follows: Millipore: Rac1 (clone 23A8, 1:1,000), RhoG (clone 1F3B3E5, 1:1,000), NG2 (AB5320, 1:200); Bethyl Laboratories: DOCK4 (A302-263A; immunoblot, 1:1,000; immunohistochemistry (IHC), 1:100), DOCK9 (A300-530A; 1:1,000); Santa Cruz: Cdc42 (sc-8401, 1:250), endomucin (clone V.7C7, 1:100), CD31 (sc-1506, 1:100), VE-cadherin (sc-6458; 1:100), EGFP (sc-8334, 1:1,000); R&D Systems: podocalyxin (AF1658, 1:100); Abcam: collagen IV (ab6586, 1:500), CA-IX (ab15086, 1:100); Sigma-Aldrich: Flag (clone M2, 1:1,000), ERK (M3807, 1:500), phospho-ERK (M8159, 1:500), DAPI (1:1,000); Cell Signaling: Cdc42 (clone 11A11, 1:1,000); Genetex: Ki67 (GTX16667, 1:100); Molecular Probes: Texas Red-conjugated phalloidin (T7471, 1:500), Alexa Fluor 488 and Alexa Fluor 546 secondary antibodies (1:300).

### Plasmids

For expression of GST–SH3 a fragment of the human DOCK4 complementary DNA corresponding to the SH3 domain (sequence shown in Supplementary Fig. 10b) was PCR amplified and subcloned into a modified pGEX4T2 vector containing AscI/PacI restriction sites. The construct harbouring GFP-tagged wild-type RhoG was a kind gift from Professor Len Stephens (the Babraham Institute, Cambridge); pEF–Flag–Dock9 was from Professor Martin Schwartz^51^; GFP-tagged DOCK4 was from Professor Hironori Katoh^17^; Flag-tagged DOCK4, DOCK4ΔDHR2 and DOCK4ΔSH3 were from Dr Vijay Yajnik^19^; CB6–EGFP–Rac wt was from Dr Michael Way (the London Research Institute); GST–ELMO was from Professor Jean-François Côté (the University of Montreal).

### siRNAs and transfection

For RNAi screens, Dharmacon (Lafayette, USA) siGENOME SMARTpool oligonucleotide duplexes were used with the exception of Rac1 for which validated SMARTpool oligo1^4^ was used. Results with siGENOME SMARTpools were validated with ON-TARGETplus individual oligonucleotides and shRNAs (Thermo Scientific, Open Biosystems). Sequences of siRNAs and shRNAs are listed in Supplementary Fig. 10. HUVEC cultured in six-well plates were transfected with 10 nM oligonucleotide duplexes using GeneFECTOR (Venn Nova, Inc.) according to the manufacturer's protocol, and HEK293T cells cultured in six-well plates were transfected with 10 nM oligonucleotide duplexes and 800 ng plasmid DNA using Lipofectamine 2000 (Invitrogen) according to the manufacturer's protocol. HEK293T cells were cultured on fibronectin-coated plates (10 μg ml^−1^; Sigma-Aldrich) for immunofuorescence and biochemical assays. HUVEC were cultured on fibronectin-coated plates for biochemical assays. Biochemical assays were performed 48 h after transfection.

### shRNAs and viral transduction

For stable knockdown HUVEC stably expressing shRNAs or empty vector EGFP control were generated by lentiviral infection in the presence of polybrene (8 μg ml^−1^). Lentiviral vectors (pGIPZ) harbouring shRNAs and EGFP were obtained from Open Biosystems. Virus production was according to http://tronolab.epfl.ch/webdav/site/tronolab/shared/protocols/LVproduction.pdf and supernatants for infection were used at 1:4 dilution of harvested virus-containing supernatants. HUVEC were infected at ∼70% confluence and used in biochemical assays 48 h after infection, or seeded onto coculture assays following fluorescence-activated cell sorting of EGFP-expressing cells. Ecotropic retrovirus producer lines used in the xenograft co-injection model were generated by transfection of TE-FLY-Mo packaging cells^15^ by calcium phosphate of retroviral vectors harbouring shRNAs, followed by selection in 0.5 μg ml^−1^ puromycin. High-titre producer clones were chosen following titration of harvested retroviruses in 3T3 fibroblasts following infection and selection with 0.5 μg ml^−1^ puromycin^15^.

### Quantitative real-time one-step PCR

Quantitative real-time PCR (RT-PCR) was performed using QuantiTect Primer Assays (Qiagen GmbH), BRILLIANT II SYBR Green QRT-PCR Master Mix KIT and the 7900HT Fast Real-Time PCR System (Applied Biosystems). Experiments shown are representative of three independent experiments.

### Pull-down assays and immunoblotting

For pull-down assays cells were grown on fibronectin-coated plates (10 μg ml^−1^). HUVEC were either cultured in 100 mm plates for 2 days to 80% confluence, serum starved in basal Angiogenesis Growth Medium (TCS CellWorks) for 5 h and stimulated with VEGF (25 ng ml^−1^); or HUVEC were transfected with siRNAs, after 24 h Human Large Vessel Endothelial Cell Growth Medium was replaced with Angiogenesis Growth Medium (TCS CellWorks) with addition of supplements and antibiotics, and after a further 24 h HUVEC were serum starved in basal Angiogenesis Growth Medium for 5 h and stimulated with VEGF (25 ng ml^−1^). HEK293T cells were transfected in 60 mm plates at 80% confluence and pull-down assays were performed 48 h after transfection. Cells were lysed in Rho lysis buffer (50 mM Tris-HCl pH 7.4, 10% Glycerol, 1% NP40, 5 mM MgCl_2_, 100 mM NaCl, EDTA-Free Complete protease inhibitors (Roche), 1 mM DTT) and pull-down assays were performed using GST–PAK1–CRIB^52^ for activated Rac1 and Cdc42, GST–ELMO1^29^ for activated RhoG or GST–SH3(DOCK4). For expression of recombinant proteins, BL21 pLYsS *Escherichia coli* were transformed with pGEX PAK1–CRIB, pGEX ELMO1, pGEX DOCK4-SH3 or pGEX empty vector and transformants were grown in 500 ml LB medium containing 100 μg ml^−1^ ampicillin (ELMO1, DOCK4-SH3 and empty vector constructs) or 100 μg ml^−1^ ampicillin and 50 μg ml^−1^ chloramphenicol (Sigma-Aldrich; PAK1–CRIB construct) until the cultures reached an OD_600_ of 0.3. Protein expression was induced with 0.3 mM IPTG (Sigma-Aldrich) for 3 h (PAK1–CRIB) or 1 mM IPTG (ELMO1, DOCK4-SH3 and empty vector) at 37 °C, bacterial cultures were pelleted, resuspended in 25 ml ice-cold TBS containing 10 mM MgCl_2_, 1 mM PMSF, 1 mM DTT and lysed by sonication on ice using a 12 mm probe (Soniprep MSE 150). Triton X-100 (10%) was added to the lysate and incubated with rocking at 4 °C for 30 min. Following centrifugation in a Sorvall RC5B, supernatants containing recombinant proteins were incubated with Glutathionine pre-coated agarose-sepharose beads (0.5 ml, GE Healthcare Bio-Sciences AB) at 4 °C for 1 h, washed and pelleted according to standard methods, and resuspended in 750 μl wash buffer. About 60 μl suspension of beads bound to the recombinant proteins were added to each pull-down assay and incubated at 4 °C for 45 min. The beads and bound protein were collected by centrifugation at 13,400*g* for 5 min, washed twice with Rho wash buffer (TBS containing 10 mM MgCl2, EDTA-free complete protease inhibitors, 1 mM DTT) and stored at −80 °C prior to analysis. Proteins in pull-down samples were resolved by SDS–polyacrylamide gel electrophoresis (PAGE). Samples were resuspended in 4 × loading buffer (NuPAGE, Invitrogen) with 50 mM DTT and heated to 70 °C for 10 mins. Polyacrylamide pre-cast gels (10%) were used for detection of proteins up to 100 kDa; 3–8% gradient polyacrylamide pre-cast gels were used for higher molecular weight proteins. Electrophoresis was performed at 150 V in either 1 × MES running buffer (Invitrogen) for 1 h or 1 × MOPS running buffer (Invitrogen) for 3 h in the presence of NuPAGE Antioxidant (Invitrogen). About 10 μl of molecular weight marker (Dual Colour Marker, Biorad 10–250 kDa) was used to track electrophoresis. The fractionated proteins were transferred to polyvinylidene fluoride filters. The Li-COR Odyssey system (Li-COR Biosciences) was used for detection and quantifications of Rac1 and RhoG; the ECL Plus detection system (GE-Healthcare-Amersham) and ImageJ software (http://rsbweb.nih.gov/ij/) were used for detection and quantification of Cdc42. Assays were repeated at least three times in independent experiments. Uncropped scans of key western blots shown in main figures are shown in Supplementary Fig. 11. For nucleotide-free pull-down assays, Rac1, RhoA and Cdc42 GST fusion proteins were prepared in the presence of 10 mM MgCl_2_ (ref. 53), and pull-down assays were performed as described above in the presence of 15 mM EDTA to chelate magnesium ions and stabilize the nucleotide-free GTPase form^51^.

### Co-IP assays

About 3 × 10^6^ HUVECs were plated onto 150 mm fibronectin-coated plates and transfected the following day with siRNAs. After 24 h, Human Large Vessel Endothelial Cell Growth Medium was replaced with Angiogenesis Growth Medium with addition of supplements and antibiotics, and after a further 24 h HUVEC were serum starved in basal Angiogenesis Growth Medium for 5 h and stimulated with VEGF (25 ng ml^−1^). Cells were lysed with 1 ml NP40 lysis buffer (20 mM Tris-HCl pH 7.5, 150 mM NaCl, 1% NP40, EDTA-free Complete protease inhibitors, 0.1% phosphatase inhibitor cocktail 2 (Sigma-Aldrich)). HEK293T cells were plated at a density of 3 × 10^6^ onto 100 mm fibronectin-coated plates and transfected the following day with siRNAs and plasmid DNA. Two days after transfection, the cells were lysed with Rho lysis buffer (50 mM Tris-HCl pH 7.4, 100 mM NaCl, 10% Glycerol, 1% NP40, 5 mM MgCl_2,_ EDTA-free Complete protease inhibitors, 0.1% phosphatase inhibitor cocktail 2). Lysates were centrifuged at 13,400*g* for 20 min and supernatants were pre-cleared by incubation with Protein G-coupled Sepharose beads (Pierce) for 30 min at 4 °C. Cleared lysates were incubated with anti-DOCK9 antibody (A300-530 A; 3 μg) for 2 h at 4 °C followed by incubation with Protein G-coupled Sepharose beads for 30 min at 4 °C. The beads were centrifuged at 13,400*g* for 2 min and washed with lysis buffer. Protein complexes bound to the beads were resolved by SDS–PAGE, and proteins were detected using the ECL Plus detection system. Quantifications were carried out using Li-COR Image Studio Lite Software (http://www.licor.com/bio/products/software/image_studio_lite/index.html).

### Mass spectrometry

A complementary DNA encoding DOCK9 protein (KIAA1058, corresponding to isoform b, NM_001130048.1) was PCR amplified and cloned into the AscI/PacI restriction sites of a Creator donor plasmid (Addgene #11690, V37 pDNR MCS SA) and recombined into a mammalian expression vector with a 3 × FLAG N-terminal tag (Addgene #11707, V180 pLP Triple-Flag SD—Acceptor). The integrity of the final construct was confirmed by sequencing. Plasmid encoding 3 × FLAG–DOCK9 or 3 × FLAG empty expression vector was transfected into HEK293T cells with a PEI-based method. Two days after transfection, the cells were washed, chilled on ice and lysed using immunoprecipitation (IP) buffer on ice containing 50 mM HEPES pH 7.5, 150 mM NaCl, 1 mM EGTA, 0.5% NP40, 1% Sodium deoxycholate, 10% Glycerol, 1.5 mM MgCl_2_, 25 mM β-glycerophosphate, 10 mM sodium pyrophosphate, 100 mM NaF, 10 μg ml^−1^ aprotinin, 10 μg ml^−1^ leupeptin, 1 μg ml^−1^ pepstatin A, 1 mM sodium orthovanadate, 1 mM PMSF, 100 nM Calyculin A and 1:1,000 dilution of Benzonase (Sigma-Aldrich). After IP with M2-agarose (Sigma-Aldrich), the beads were washed three times with ice-cold IP buffer and once with ice-cold 200 mM ammonium bicarbonate. Proteins were eluted from the beads with 50 mM phosphoric acid, pH 2.5 and subjected to solid-phase (SCX) trypsin digestion with reduction and S-alkylation with iodoacetamide. The peptides were separated and analysed by LC-MS/MS using an Orbitrap Elite mass spectrometer. MASCOT was used to identify proteins from the MS/MS peak lists.

### Organotypic angiogenesis assay and viral transduction

In the organotypic angiogenesis assay^4^, 8.5 × 10^3^ HUVECs transfected with siRNAs or infected with lentiviruses were seeded onto CFs that had been plated at 2 × 10^4^ cells onto 24-well plates and grown to confluency over 7 days. Seeding was 18 h after HUVEC transfection or infection, in 50:50 Large Vessel Endothelial Cell Growth Medium and DMEM 10% FCS, which was replenished every 2 days. Tubule formation was assessed 5 days after seeding by CD31 staining or by visualization of EGFP-expressing HUVEC; where indicated the coculture media were replenished with media containing VEGF (25 ng ml^−1^; Sigma-Aldrich) up to 48 h prior to imaging and without prior media change. For lumen assays, the culture media were changed to Angiokit Optimized Growth Medium at 9 days after seeding HUVECs onto CFs. Treatment of the cocultures with LatB (Santa Cruz) was at 0.01 μg ml^−1^. Treatment with the ROCK inhibitor Y27632 (Tocris) was at 10 μM^4^. Treatment schedules are shown in Supplementary Fig. 1a.

### Immunostaining and imaging

HUVEC-HDF cocultures^15^ for confocal imaging were grown on glass bottom dishes (Mat Tek Corporation). For visualization of lumens, the cultures were fixed in 4% paraformaldehyde, washed and permeabilized with 0.1% Triton X-100, followed by blocking in 0.5% BSA. Collagen IV (1:500) and podocalyxin (1:100) antibodies were applied at 4 °C overnight, secondary antibodies conjugated with Alexa Fluor 488 and Alexa Fluor 546 (1:1,000) were applied for 1 h. Filopodia protruding from tubules with lentiviral EGFP expression were visualized in live cultures using confocal microscopy. Unless otherwise stated, confocal images of filopodia and lumens are maximum intensity projections of Z-stacks (15 sections of 1 μm thickness) obtained using a Zeiss LSM710 inverted confocal microscope controlled by the Zen acquisition software using a × 40 oil immersion Plan-Apochromat lense; or a Nikon A1R confocal microscope controlled by NIS-Elements C software using a × 40 oil immersion CFI S.Fluor lense. Filopodia in confocal images (maximum intensity projections) or phase contrast images were counted manually with counts corroborated by two observers; three or four images of tubules 400–500-μm long with identifiable tips were quantified for each organotypic coculture. Quantification of lumens was from maximum intensity projections of Z-stacks; lumen length was identified by apical podocalyxin staining^37^; four or more images of established tubes of ∼250–500 μm individual length were quantified for each organotypic coculture; sprouts<170 μm were excluded from the analysis. Confocal Z-stacks were 3D-rendered using the Visualization module of Volocity software, except for the 3D movies that were performed using the automatic surface rendering function of Imaris 7.5 software (Bitplane AG, Switzerland). Staining of tumour sections was as described above using endomucin (1:100) and CA-IX (1:200) antibodies applied overnight at 4 °C.

IHC of cocultures was performed using a mouse anti-human CD31 Tubule Staining Kit (TCS CellWorks) according to the Angiokit protocol (TCS CellWorks). Phase contrast images of cocultures stained for CD31 by IHC were obtained with a Nikon light microscope using a × 4 objective, or an Olympus CKX41 inverted microscope using a × 40 objective for filopodia visualization. Quantification of tubules, branches and total tubule length was from immunohistochemical images using the Angiosys software (six or more images were quantified for each organotypic coculture) or from immunofluorescence images manually using the Volocity software for determination of tubule length.

### Time-lapse microscopy and cell tracking

Multisite time-lapse microscopy of HUVEC stably expressing EGFP was performed in a humidified, CO_2_-equilibrated chamber using a Diaphot inverted microscope (Nikon, Kingston upon Thames, UK) equipped with a motorized stage (Prior Scientific, Oxford, UK)^4^. Confocal time-lapse movies of filopodia were generated using a motorized stage and the Zeiss Zen software. Images shown of the time-lapse movies are representative of the experiments quantified. Cell spreading and bipolarity were assessed using the image processing and analysis modules of Simple PCI. Displacement rates were determined using the manual tracking modules of Volocity and ImageJ software.

### Generation of *Dock4* null mice

The *Dock4* null mouse line was generated using gene targeted C2 ES cells (Clone N01793P1_W109G2) obtained from Norcomm, part of the International Knockout Mouse Consortium. Southern blotting was performed to confirm the *Dock4* targeted allele using a 720 bp probe located 5′ of the targeting vector homology region. The ES cells were then microinjected into CD-1 blastocysts to generate chimaeras and these mice mated with C57BL/6J to effect germline transmission of the mutant allele. Heterozygous mice were incrossed and pregnant dams killed for analysis of embryos. Genotyping was by PCR using the following primer pairs:

WT Band—Dock4_5′F (5′-CACACACCTGCTACATCATGC-3′) and Dock4_5′ WTR (5′-TTTACCTCCCTGTGCTCCAC-3′); KO Band—Dock4_5′F and Dock4_5′KOR (5′-CATTGGTGAGCAGAGCCTTCG-3′).

All animal use was authorized by the University of Leeds Animal Ethics Committee and by the Home Office, UK and experiments were performed according to Home Office Regulations and the CCCR guidelines. All animals were maintained in Optimice individually ventilated cages (Animal Care Systems) at 21 °C, 50–70% humidity, light/dark cycle 12:12 h on RM1 diet (Special Diet Services, Witham, UK) *ad libitum* and bedding of Pure ‘o Cell (Datesand, Manchester, UK) with enrichment of Sizzlenest (International Product Supplies, London, UK).

### Embryo analysis

Serial transverse sections (4 μm) of formalin-fixed E13.5 embryos were cut using a microtome. Slides were dried overnight at 37 °C and heated for 20 min at 60 °C. Haematoxylin and eosin staining of every tenth section was used to select the anatomical level according to the ref. 54. IHC for CD31, NG2 and DOCK4 was carried out using standard methods. Images were captured using an Axioplan Zeiss microscope fitted with AxioCam color camera and AxioVision 4.6 software (Carl Zeiss). Images at the brain level were captured using a × 20 objective and at the lung level using a × 4 objective. Vessel diameter for each lumen was defined, and the best fit circle for each lumen was determined using the AxioVision software (smallest identifiable lumens were 8 μm).

### *In vivo* tumour models

About 1x10^6^ BE cancer cells were co-injected s.c. with 10 × 10^6^ retrovirus producers irradiated at 20 Gy in the flanks of 6–8-weeks-old MF1 nude mice. Tumours were excised when they reached 1.3 cm diameter, fixed in 4% paraformaldehyde and cryosections (10 μm) were immunostained. Images were acquired using Zeiss 710 inverted confocal microscrope with a × 20 Plan-Apochromat dry lens. For CA-IX a × 20 objective and motorized stage were used to acquire 3 × 3-tiled images (Zeiss Zen software). Lumen and total vessel numbers were counted using the ImageJ cell counter plugin. For the intracranial model, 1 × 10^5^ EO771 cells were injected stereotactically into the striatum of 9–10-week-old *Dock4*^*−/+*^ and WT siblings (three females each). Mice were culled and tumours excised on presentation of neurological symptoms in the first mouse, fixed as described for s.c. tumours. Images of sections across whole tumours were generated using tile-scanning of 6 × 6 Z-stacks (compiled from 5 μm slices using NIS-Elements AR software) acquired with a Nikon A1R confocal microscope, × 10 objective. Lumen width was measured using Volocity as the shortest representative measurement across the lumen. Lumens were defined as visible gaps surrounded by endomucin staining on sections imaged as above (smallest identifiable lumens were 15 μm). Image file names were allocated random numbers and analysed blind.

### Cancer patient samples

Specimens were from Leeds Teaching Hospital NHS Trust patients under Leeds research ethics committee approval. Informed consent was obtained from all participants.

### Statistical analyses

Data are presented as mean values with s.e. generated by independent repeats of experiments. *P* values were obtained from *t-*tests with unpaired samples except for analysis of the pull-down assays where a paired test was used. *P* values of <0.05 represent statistically significant differences.

## Additional information

**Accession codes**: Proteomics dataset files can be accessed at the massive server (massive.ucsd.edu). MassIVE ID: MSV000079100.

**How to cite this article:** Abraham, S. *et al.* A Rac/Cdc42 exchange factor complex promotes formation of lateral filopodia and blood vessel lumen morphogenesis. *Nat. Commun.* 6:7286 doi: 10.1038/ncomms8286 (2015).

## Supplementary Material

Supplementary Figures and Tables Supplementary Figures 1-11, Supplementary Tables 1-3.

Supplementary Movie 1 DOCK4 is necessary for lumen formation (related to Figure 1e). Imaris 3D reconstruction of control tubules at 14 days after seeding onto CFs HUVEC infected with an empty EGFP lentivirus. Cocultures were stained for the luminal marker podocalyxin and basement membrane marker collagen IV. Note luminal localization of podocalyxin. Movie is control for Supplementary Movie 2.

Supplementary Movie 2 DOCK4 is necessary for lumen formation (related to Figure 1e). Imaris 3D reconstruction of tubules at 14 days after seeding HUVEC with DOCK4 knockdown (lentiviral shRNA1) onto CFs. Cocultures were stained as in Supplementary Movie 1. The reconstruction shows thin tubules lacking lumens with podocalyxin distributed diffuse in the cytoplasm.

Supplementary Movie 3 DOCK4 is required for lateral protrusions and tubule remodeling (corresponds to Figure 1f). Tubule remodeling by HUVEC infected with empty EGFP lentivirus at 4 days after seeding onto CFs. Movie length, 35 h; total of 36 frames (1 frame/h). Movie is control for Supplementary Movie 4.

Supplementary Movie 4 DOCK4 is required for lateral protrusions and tubule remodelling (corresponds to Figure 1f). Tubules formed by HUVEC with DOCK4 depletion (lentiviral shRNA1) seeded onto CFs as in Supplementary Movie 3. Note that tubules elongate but lack lateral protrusions in the absence of DOCK4. Movie length, 35 h; total of 36 frames (1 frame/h).

Supplementary Movie 5 DOCK4 is required for lateral protrusions but is dispensable for anastomosis (corresponds to Supplementary Figure 1j). Control for Supplementary Movie 6. Tubule remodelling and anastomosis by HUVEC infected with empty EGFP lentivirus at 5 days after seeding onto CFs. Movie length, 36 h; total of 36 frames (1 frame/h). Movie is control for Supplementary Movie 6.

Supplementary Movie 6 DOCK4 is required for lateral protrusions but is dispensable for anastomosis (corresponds to Supplementary Figure 1j). Tubule formation by HUVEC with DOCK4 depletion (lentiviral shRNA1) seeded onto CFs as in Supplementary Movie 5. Note that anastomosis proceeds in absence of DOCK4. Movie length, 36 h; total of 36 frames (1 frame/h).

Supplementary Movie 7 Control of protrusive activity and remodelling by Rac1 (corresponds to Supplemental Figure 2b). Tubule formation by HUVEC infected with an empty EGFP lentivirus seeded onto CFs as in Supplementary Movie 5. Movie length, 28 h; total of 28 frames (1 frame/h). Movie is control for Supplementary Movie 8.

Supplementary Movie 8 Control of protrusive activity and remodelling by Rac1 (corresponds to Supplemental Figure 2b). Tubule formation by HUVEC with Rac1 depletion (lentiviral shRNA1) seeded onto CFs as in Supplementary Movie 5. Note blockade of tubule remodeling in the absence of Rac1. Movie length, 28 h; total of 28 frames (1 frame/h).

Supplementary Movie 9 DOCK4 controls lateral filopodia (corresponds to Figure 3d) Filopodial protrusions on tubules formed by HUVEC expressing EGFP at 7 days after seeding onto CFs. Note filopodia extending and retracting dynamically at proximal and lateral sites. Movie length, 15 min; total of 15 frames (1 frame/min). Movie is control for Supplementary Movie 10.

Supplementary Movie 10 DOCK4 controls lateral filopodia (corresponds to Figure 3d) Filopodial protrusions on tubules formed by HUVEC with DOCK4 depletion (shRNA) seeded onto CFs as in Supplementary Movie 9. Note that proximal filopodia persist in the absence of DOCK4. Movie length, 15 min; total of 15 frames (1 frame/min).
