# Supplementary material for: A Rac/Cdc42 exchange factor complex promotes formation of lateral filopodia and blood vessel lumen morphogenesis
Source: Nat Commun. 2015 Jul 1;6:7286. doi: 10.1038/ncomms8286 (PMC4507007; doi:10.1038/ncomms8286)
Supplement: Supplementary Figures and Tables — Supplementary Figures 1-11, Supplementary Tables 1-3. [file ncomms8286-s1.pdf]

Supplementary Figure 1

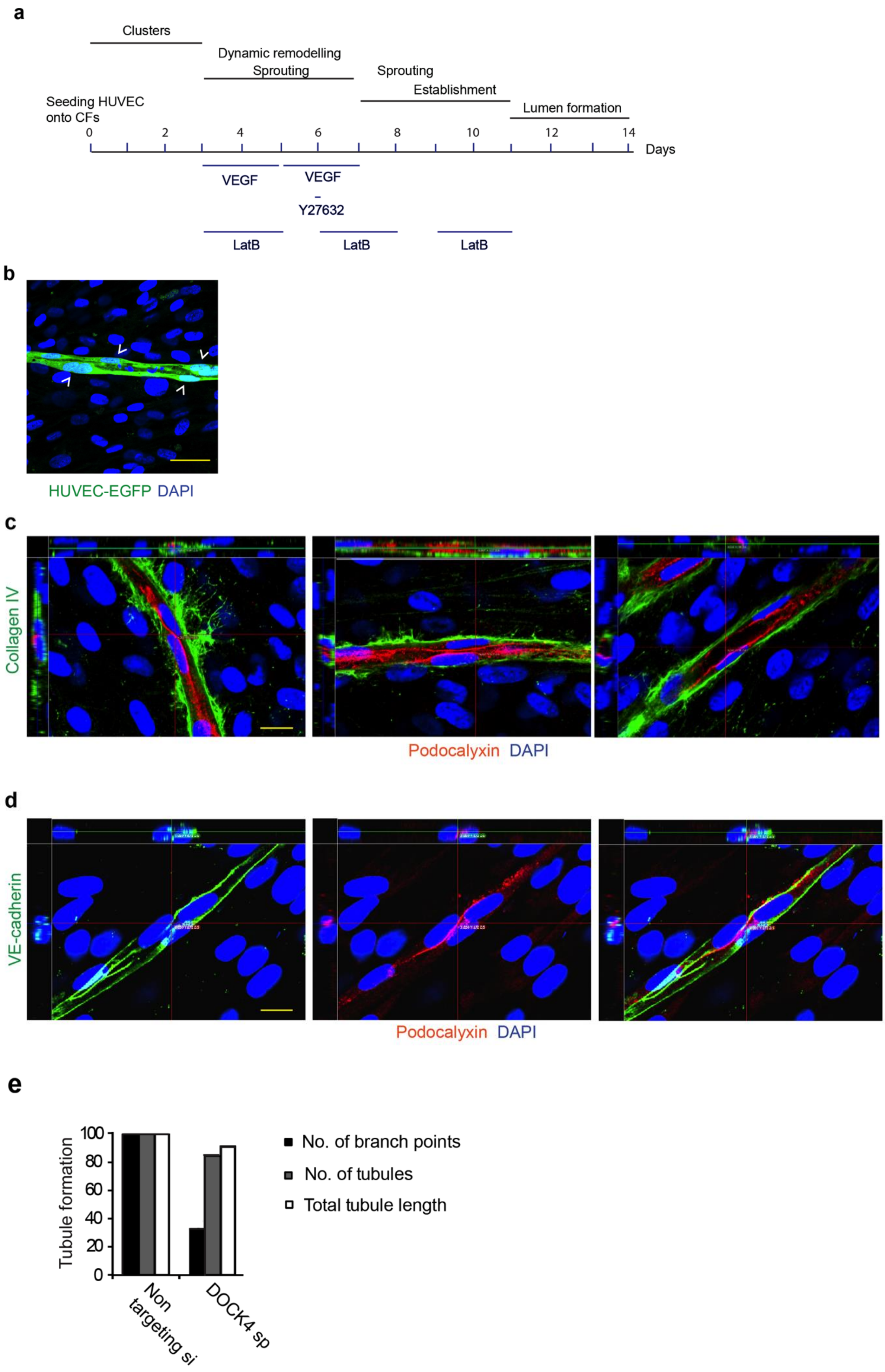

Supplementary Figure 1

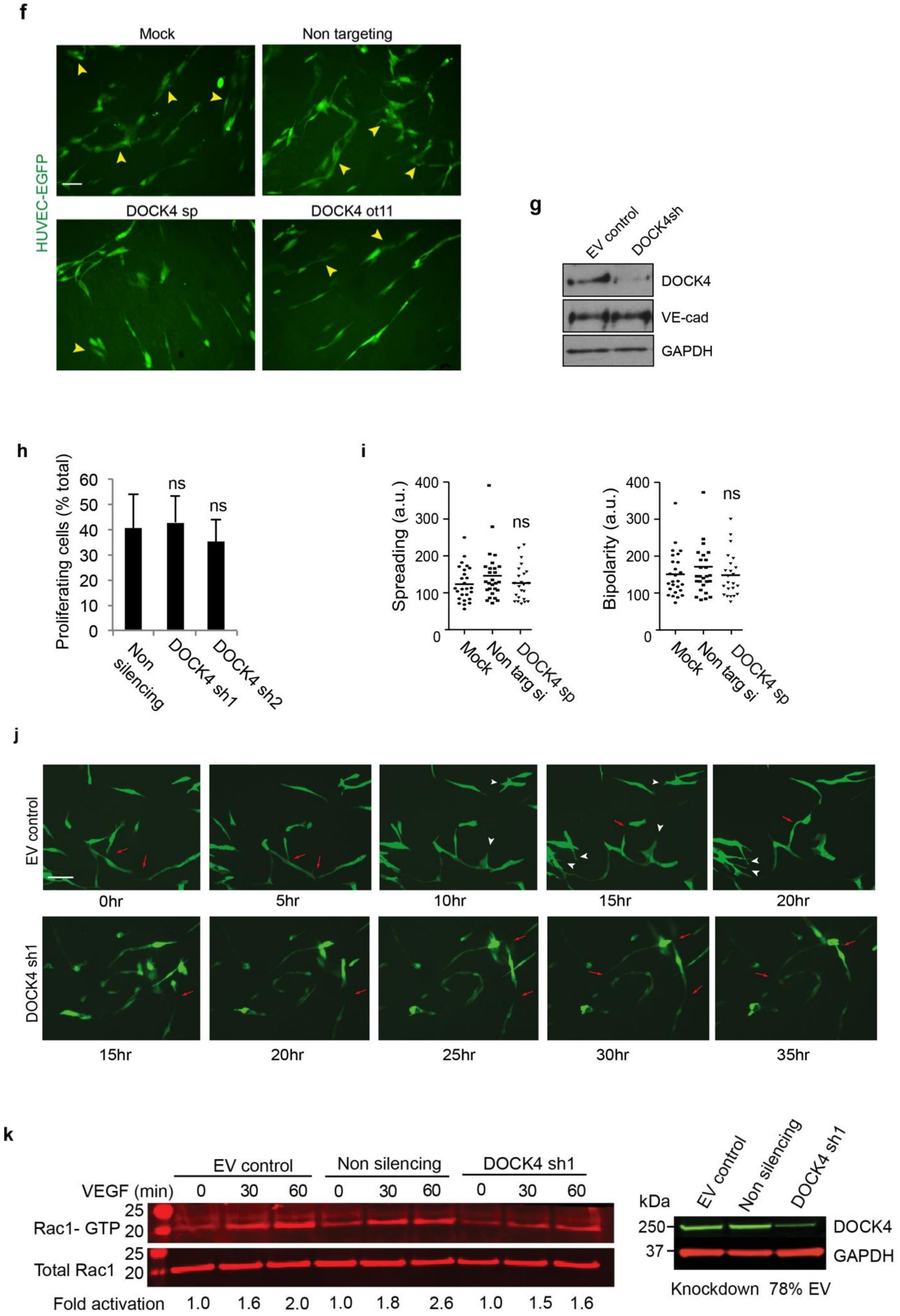

l

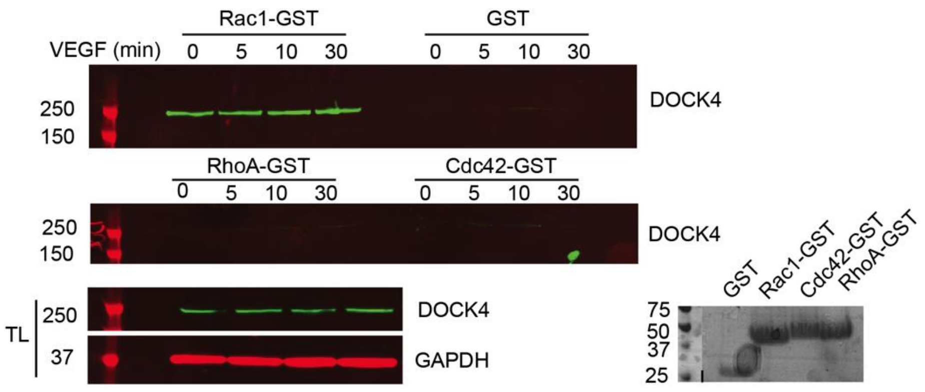

Supplementary Figure 1. Rac GEF DOCK4 controls clusters and protrusive activity. (**a**) Stages of tubule morphogenesis in the coculture system and treatment schedules. (**b**) Onset of lumen formation at 11d after seeding of HUVEC-EGFP onto CFs. Note the presence of apposing endothelial cells (white arrows) around the developing lumen. Scale bar 50µm. (**c**) Confocal images of developing tubes 14d after seeding HUVEC onto CFs show apical localization of podocalyxin; collagen IV marks basement membrane. Scale bar 15µm. (**d**) Lateral adhesions at onset of lumen formation visualized by VE-cadherin staining. Scale bar 15µm. (**e**) Histogram: total tubule length, tubules and branch points 5d after seeding HUVEC with DOCK4 depletion (sp, smartpool siRNA) onto CFS; *n*= 18 microscopic fields from three organotypic cocultures, representative experiment from two rounds of screens. (**f**) Endothelial cell clusters (yellow arrowheads) 2d after seeding HUVEC-EGFP with DOCK4 depletion (sp, smartpool siRNA; ot, on-target plus siRNA) onto CFs. Scale bar 100µm. (**g**) VE-cadherin immunoblot following DOCK4 depletion in HUVEC (sh, shRNA1). (**h**) Histogram: endothelial cell proliferation by Ki67 staining 2d after seeding HUVEC with DOCK4 depletion (sh, shRNA) onto CFs. Values represent Ki67 positive cells as percentage of total ± s.e.m.; *n*=12 microscopic fields (20x objective), representative experiment. Knockdown: 76.6% reduction compared to non silencing control. ns, non significant by two-tailed *t* test compared to non silencing control. (**i**) Scatter plot: spreading and bipolarity following DOCK4 depletion (sp, smartpool siRNA) of HUVEC-EGFP 3d after seeding onto CFs; n=24 cells from four organotypic cocultures; knockdown: 84.0% reduction compared to non targeting. ns, non significant by two-tailed *t* test compared to non targeting. (**j**) Still images from Supplemental movies S5 and S6 of tubule formation following DOCK4 depletion (shRNA, sh; EV, empty vector) 5d after seeding HUVEC onto CFs. White arrowheads: protrusions persisting over 5hrs; red arrows: anastomoses. Scale bar 50µm. (**k**) Left panel: immunoblot of Rac1 activation on VEGF stimulation (25ng/ml) in HUVEC following DOCK4 depletion (shRNA, sh; EV, empty vector); fold activation (Rac1-GTP/ total Rac1) compared to unstimulated controls. Right panel: DOCK4 knockdown by immunoblot. (**l**) Immunoblots of DOCK4 in GST-Rac1, RhoA and Cdc42 pulldowns in HUVEC. Bottom left panels: total lysate (TL); bottom right: GST proteins in pulldowns.

Supplementary Figure 2

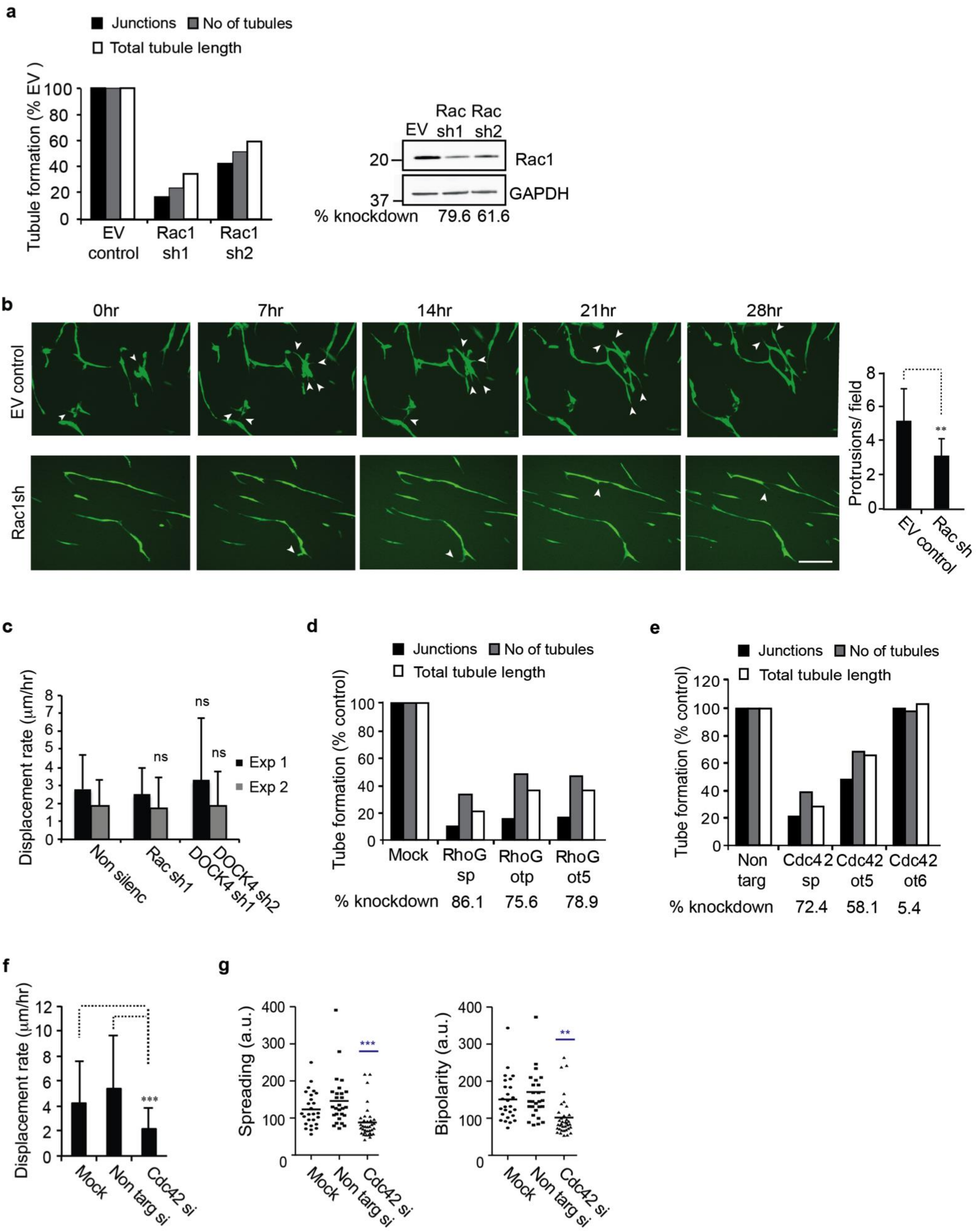

Supplementary Figure 2. RhoGTPases required for angiogenesis. (**a**) Quantification of tubule formation 7d after seeding HUVEC with Rac1 depletion (shRNA, sh) onto CFs (*n*=12 microscopic fields), representative experiment. (**b**) Still images from Supplemental movies S7 and S8 of HUVEC with Rac1 depletion in **a**. Arrowheads point to protrusions persisting for over 5hrs. Histogram: quantification over 48hrs of protrusions persisting for over 5 hrs. (**c**) Histogram: quantification of displacement rate (linear distance travelled) over 24hrs following Rac1 or DOCK4 depletion (shRNA, sh) starting at 3d after seeding onto CFs; error bars indicate s.d.; *n*= number of single cells tracked in two experiments (exp 1: non silencing, *n*=61; Rac sh1, *n*=45; DOCK4 sh1, *n*=54; exp 2: non silencing, *n*=32; Rac sh1, *n*=31; DOCK4 sh2, *n*=42) from timelapse movies (exp 1: non silencing, 8; Rac sh1, 8; DOCK4 sh1, 10; exp 2: non silencing, 5; Rac sh1, 6; DOCK4 sh2, 7). (**d, e**) Quantification of tubule formation after RhoG (**d**) or Cdc42 (**e**) depletion (sp, smartpool siRNAs; ot, on-target plus siRNAs) in HUVEC 5d after seeding onto CFs, (*n*= 12 microscopic fields), representative experiment. (**f**) Histogram: quantification of displacement rate as in **c** following Cdc42 depletion (sp, smartpool siRNA) starting at 2d after seeding onto CFs; error bars indicate s.d.; *n*= 24 single cells tracked from 4 movies. (**g**) Scatter plot: quantification of cell spreading and bipolarity following Cdc42 depletion (sp, smartpool siRNA; ot, on-target plus siRNA) of HUVEC-EGFP at 3d after seeding onto CFs; error bars indicate s.d.; *n*= 24 single cells from four cocultures. ** $P<0.01$, *** $P<0.001$ by two-tailed *t* test.

Supplementary Figure 3

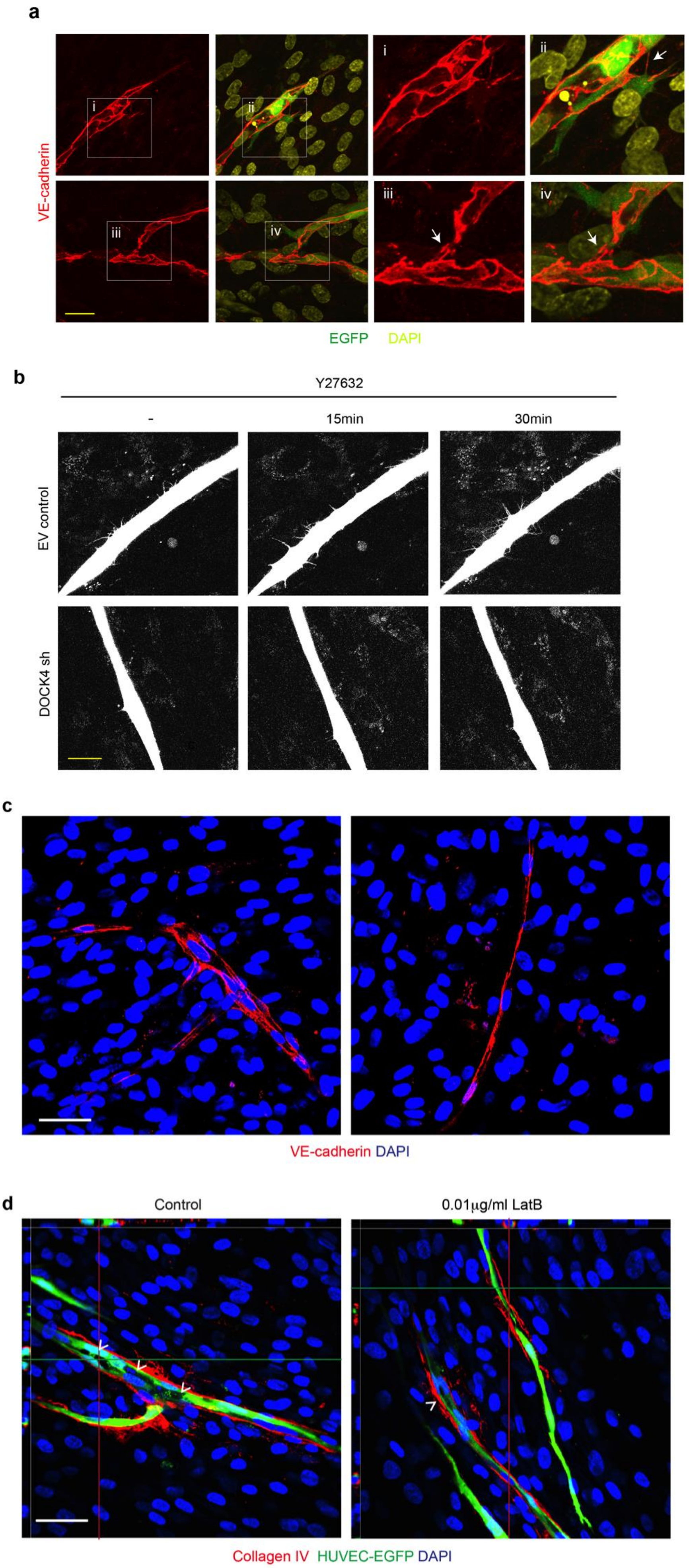

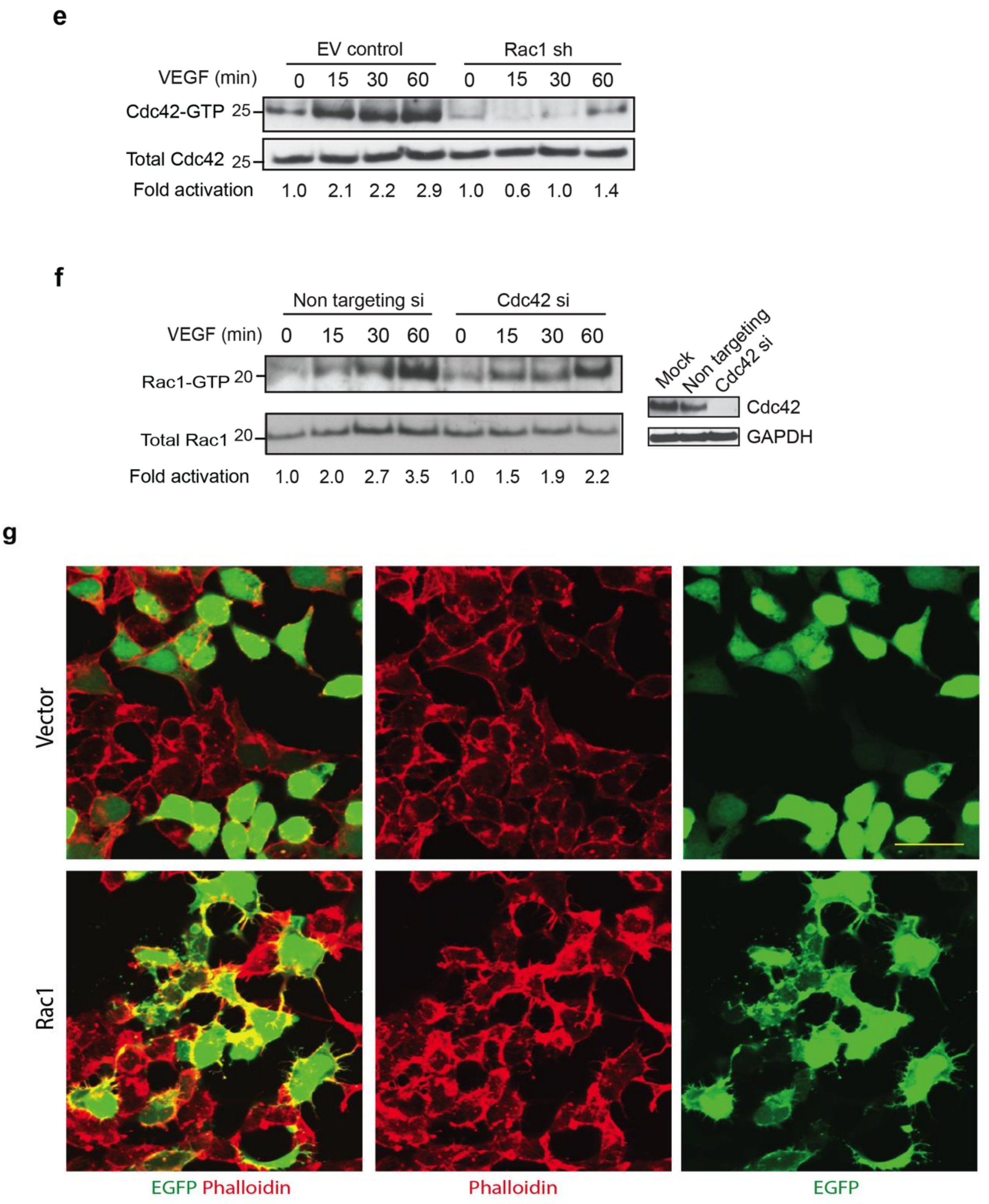

Supplementary Figure 3. Rac1 controls Cdc42 activation and filopodia formation. (**a**) VE-cadherin localization at filopodia (white arrows) in HUVEC-EGFP on 24 hr VEGF treatment (25ng/ml) 6d after seeding HUVEC onto CFs. White arrows point to VE-cadherin localization at filopodia at sites of cell-cell contact. (**b**) Filopodia blockade with DOCK4 depletion (shRNA1, sh; EV, empty vector) persists on Y27632 treatment. Tubules were treated with Y27632 at 6d after seeding onto CFs for the indicated timepoints. Scale bar 50μm. (**c-d**) Appearance of tubules and adherens junctions on treatment with 0.01μg/ml Latrunculin B (LatB). Cocultures were treated with 0.01μg/ml LatB for 48hrs at 3d, 6d and 9d after seeding HUVEC onto CFs (6 day total treatment), fixed and stained for VE-cadherin to mark adherens junctions and collagen IV to mark the basement membrane. Note the lack of sprouts and thinner appearance of treated tubules compared to controls in **c**; treatment allowed junction formation but resulted in fewer lateral cell-cell contacts. White arrowheads

in **d** point to sites of lumen formation. (**e**) Immunoblot of Cdc42 activation on VEGF stimulation (25ng/ml) in HUVEC following Rac1 depletion (shRNA, sh; EV, empty vector); fold Cdc42 activation (Cdc42-GTP/ total Cdc42) is compared to unstimulated controls. (**f**) Immunoblot of Rac1 activation on VEGF stimulation (25ng/ml) in HUVEC following Cdc42 depletion (siRNA, si); fold Rac1 activation (Rac1-GTP/ total Rac1) is compared to unstimulated controls. (**g**) Images of phalloidin stained 293T cells following overexpression of EGFP-Rac1 or Vector (EGFP) corresponding to Figure 3h. Scale bar, 50μm.

Supplementary Figure 4

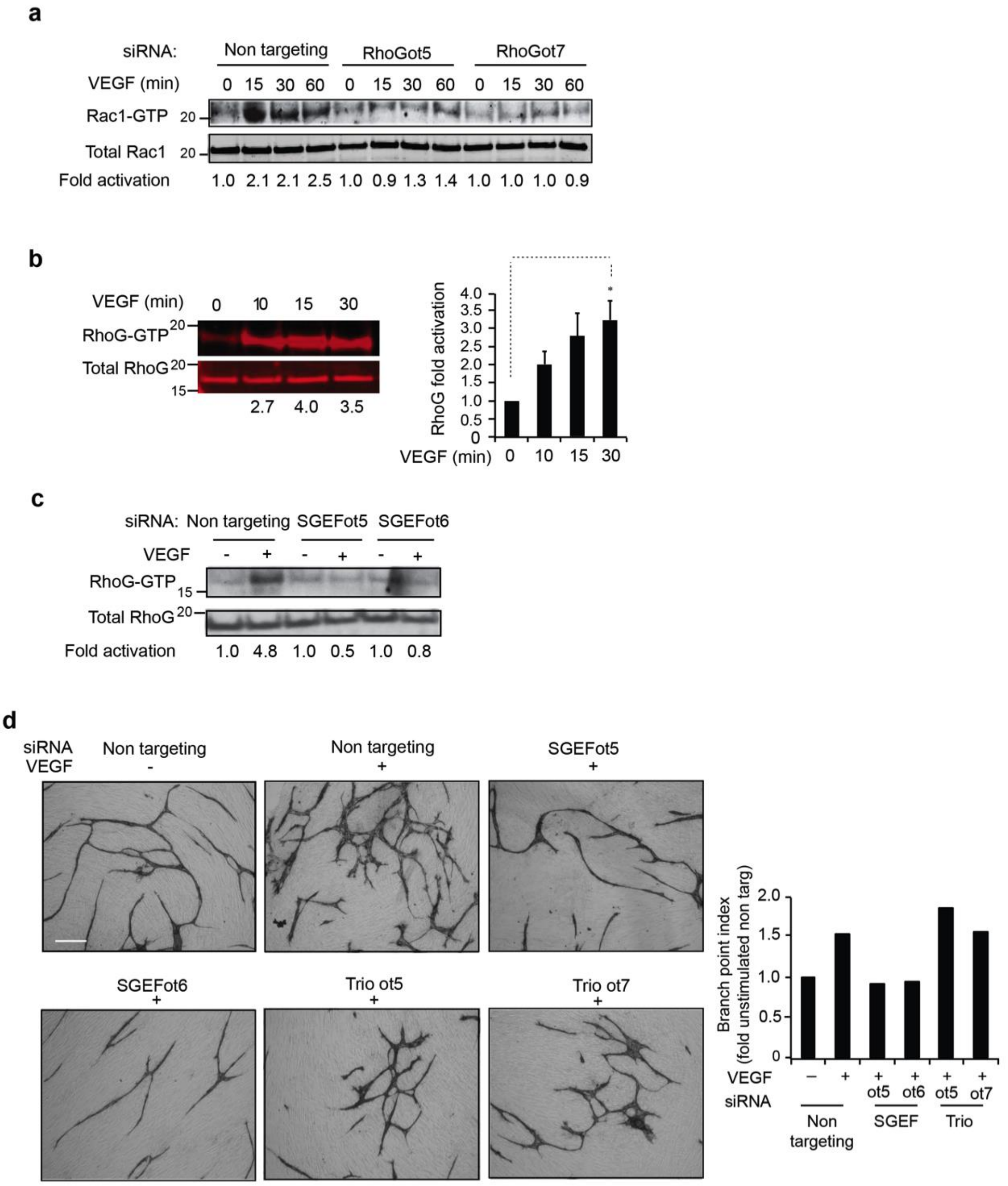

Supplementary Figure 4. SGEF driven RhoG activation regulates Rac1 activation downstream of VEGF. (**a**) Immunoblot of Rac1 activation (Rac-GTP/ total Rac) on VEGF stimulation (25ng/ml) in HUVEC following RhoG depletion (ot, on-target plus siRNAs). (**b**) Immunoblot of RhoG activation on VEGF stimulation (25ng/ml) in HUVEC. Histogram shows fold RhoG activation (GTP-bound RhoG/ total RhoG) compared to unstimulated control; error bars s.e.m. (*n*= 3 independent experiments). (**c**) Immunoblot of RhoG activation on 30min VEGF stimulation (25ng/ml) in HUVEC after SGEF depletion (ot, on-target plus siRNAs); fold RhoG activation (RhoG-GTP/ total RhoG) compared to unstimulated controls. (**d**) Images of organotypic cocultures (CD31 staining) treated with VEGF (25ng/ml) and following depletion of SGEF or Trio (ot, on-target plus siRNAs) in HUVEC 5d after seeding onto CFs. Scale bar, 200μm. Histogram: branch point index (*n*= 12 microscopic fields from three cocultures, representative experiment).

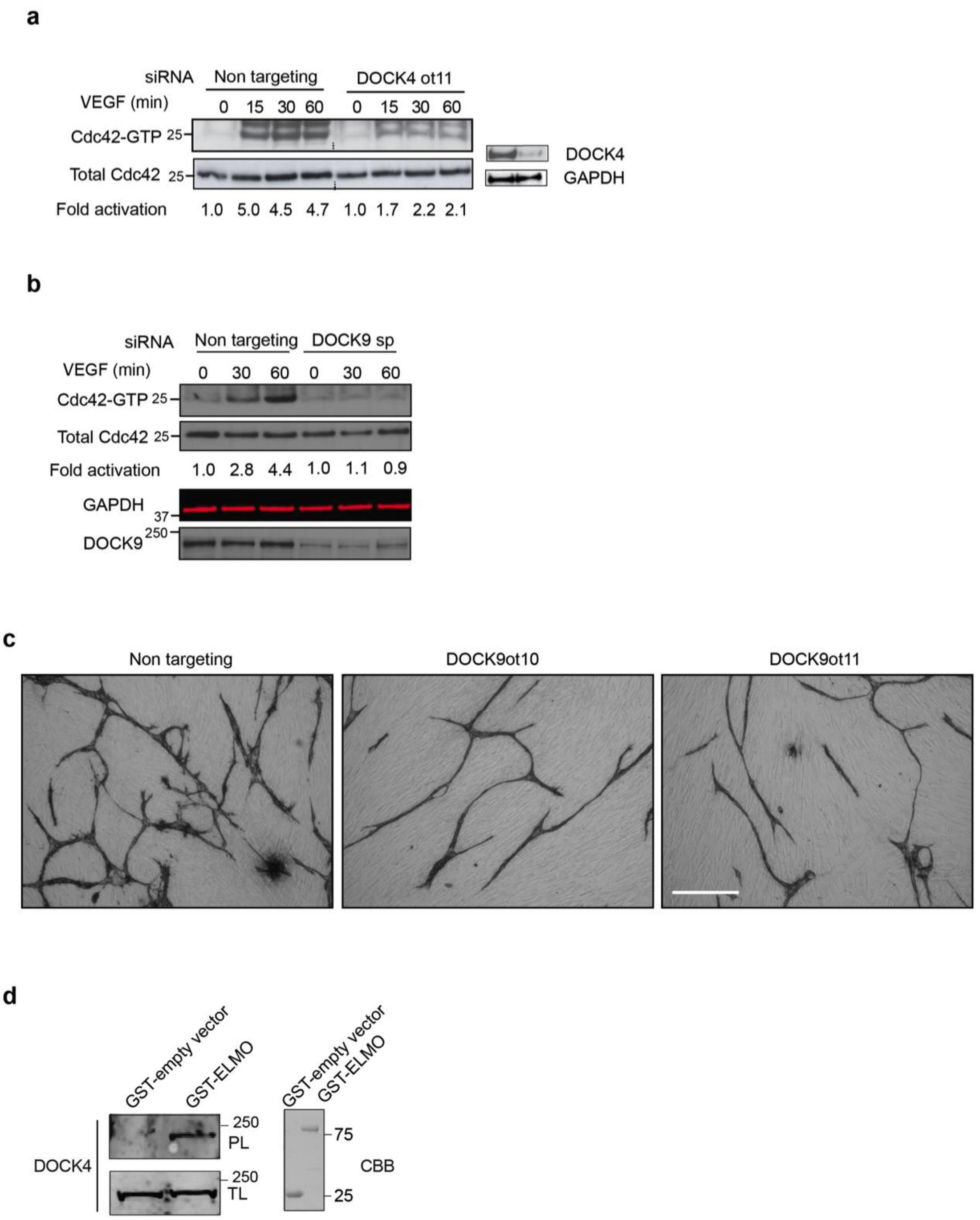

Supplementary Figure 5. DOCK4 and DOCK9 control Cdc42 activation and interaction of DOCK4 with ELMO. (**a**) Immunoblot of Cdc42 activation following DOCK4 depletion (ot, on-target plus siRNA); fold Cdc42 activation (Cdc42-GTP/ total Cdc42) compared to unstimulated controls. Knockdown: 80.6% reduction compared to the non targeting control. (**b**) Immunoblot of Cdc42 activation (Cdc42-GTP/total Cdc42) on VEGF stimulation (25ng/ml) in HUVEC following depletion of DOCK9 (smartpool siRNA, sp). (**c**) Images of organotypic cocultures (CD31 staining) treated with VEGF (25ng/ml) following depletion of DOCK9 (ot, on-target plus siRNAs) in HUVEC 5d after seeding onto CFs. Scale bar, 200μm. (**d**) Pulldown assay shows interaction of DOCK4 with RhoG effector ELMO in 293T cells.

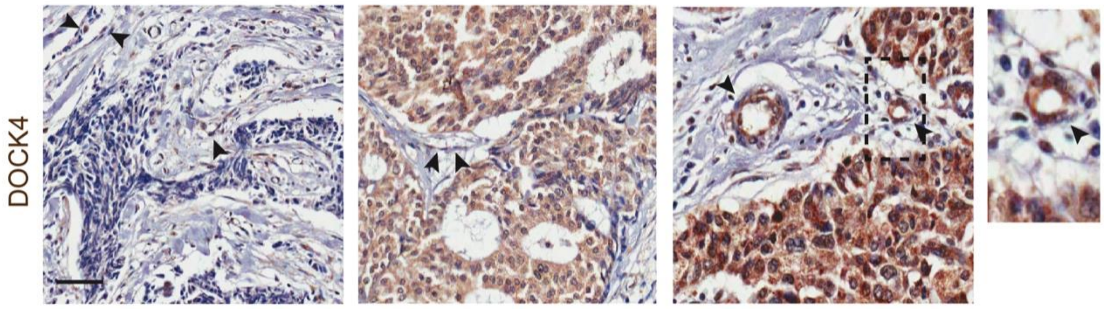

Supplementary Figure 6. DOCK4 expression in patient tumours. DOCK4 expression in blood vessels (black arrowheads) of ductal breast carcinoma specimens showing different levels of DOCK4 expression in the tumour cell compartment and blood vessels. Scale bar, 200μm.

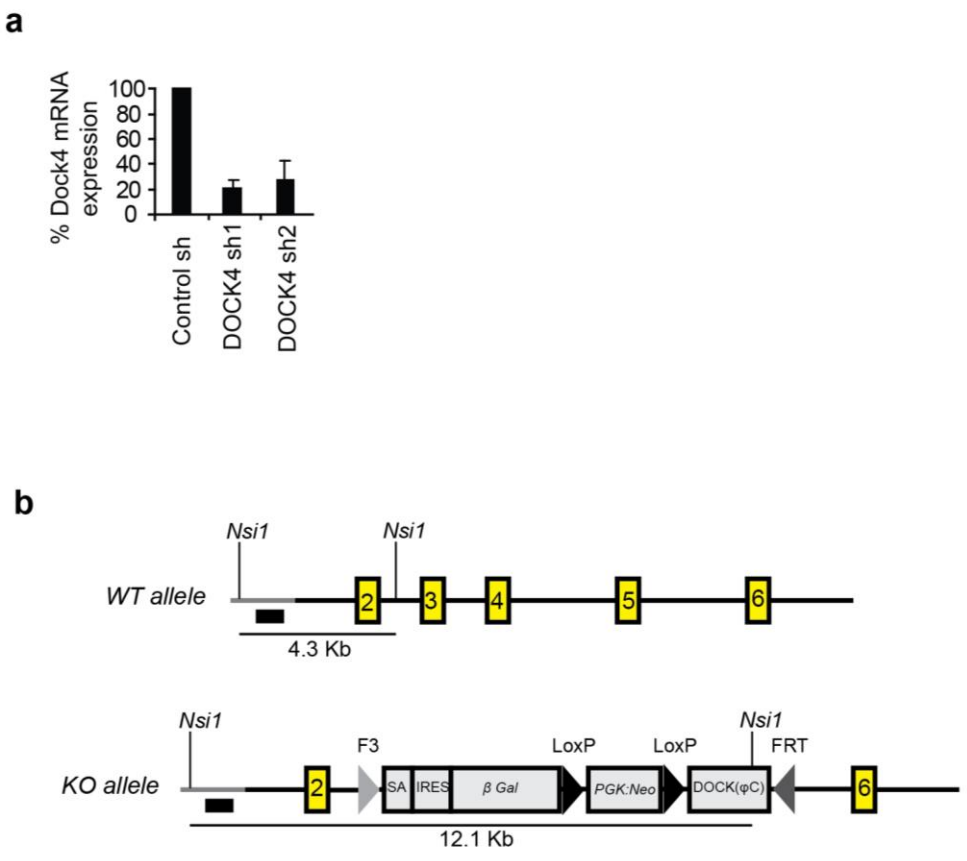

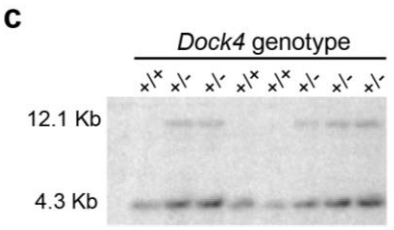

d

	***Dock4* WT**	***Dock4* Het**	***Dock4* KO**
**E11.5**	6	9	0
**E10.5**	2	6	0
**E9.5**	0	6	0
**E8.5**	5	14	0
**E7.5**	2	13	0

Supplementary Figure 7. Retroviruses used in the *in vivo* tumour co-injection model and generation of the *Dock4* null heterozygous mouse line. (**a**) Ecotropic retroviruses harbouring DOCK4 shRNAs were tested in NIH3T3 fibroblasts. Histogram: shRNA-mediated DOCK4 knockdown determined by qPCR. (**b**) Schematic showing the wild type (WT) and the targeted knockout (KO) *Dock4* alleles. In the targeted allele, exons 3-5 were replaced by the targeting cassette for frameshift of the open reading frame. Yellow boxes show exons, main black lines show homology regions, grey lines show homology outside of the targeting vector. SA = Splice Acceptor, IRES = Internal Ribosomal Entry Site. Black boxes show position of the Southern probe that detects bands shown upon NsiI digestion. (**c**) Southern blot of tail DNA from an F1 litter of *Dock4* mice derived from a chimaera crossed to C57BL/6J females. + = WT allele, - = KO allele. (**d**) Table shows numbers of embryos analysed from *Dock4*+/- incrosses. Note the absence of *Dock4* null homozygous embryos.

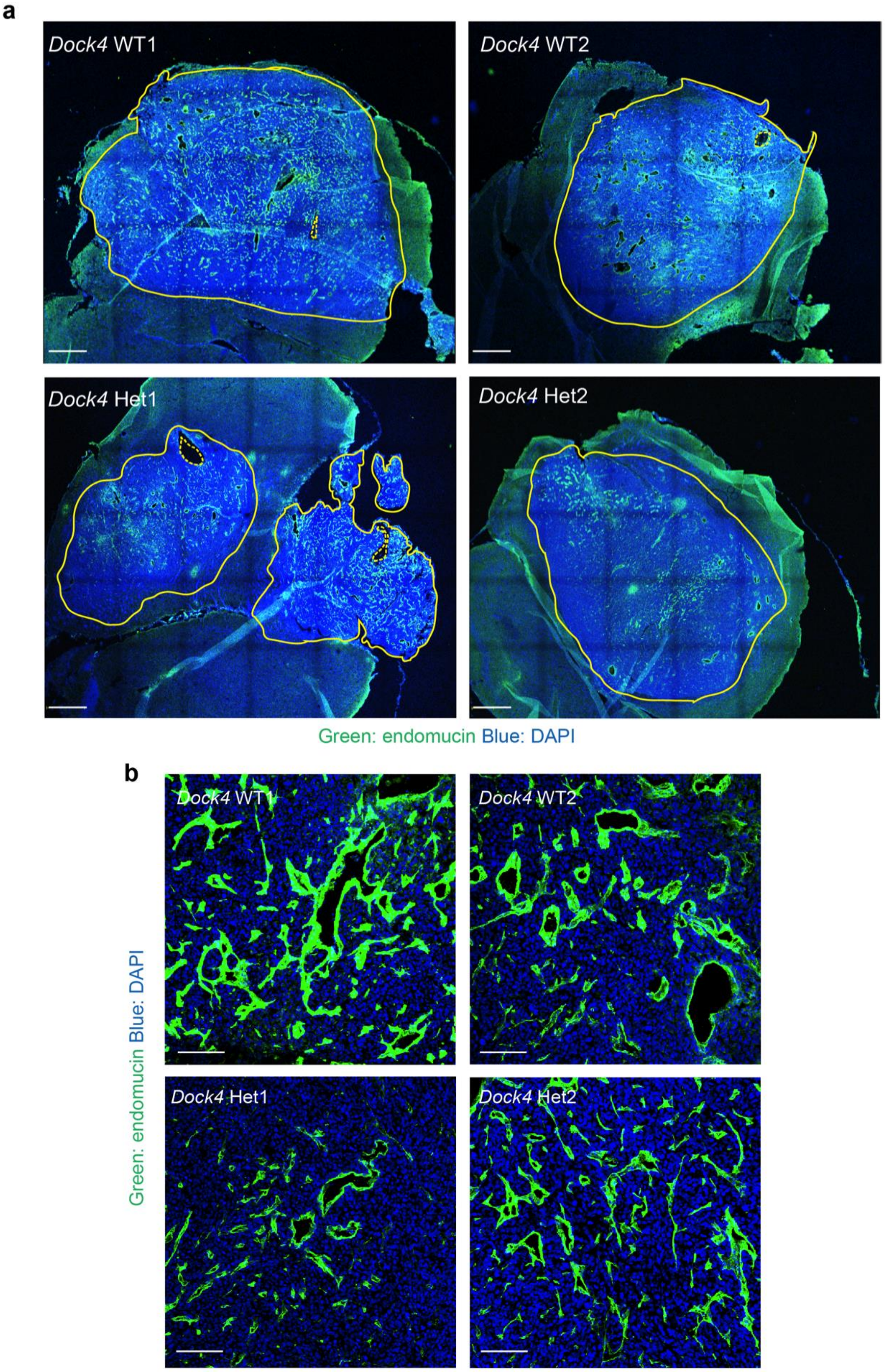

Supplementary Figure 8. DOCK4 controls blood vessel lumen calibre in tumours. (**a**) Examples of tiled images of intracranial tumours used for lumen analysis. (**b**) Confocal images obtained with a 20x objective representative of tumour areas with lumenized blood vessels.

a

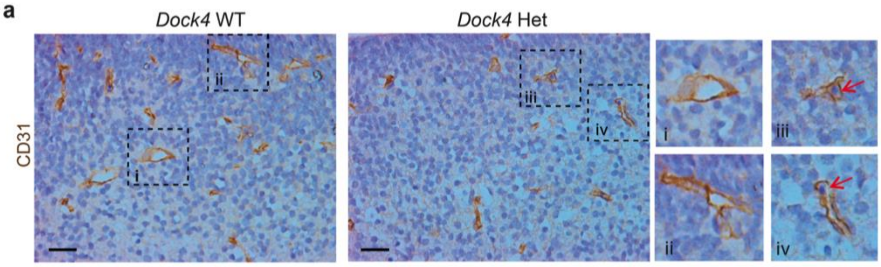

Lumen width analysis				
	Average lumen width (μm)	Frequency (%) of lumen width (μm)		
		8.0-14.0	14.0-20.0	>20
Dock4 WT	18.6 (±3.5)	10.0	59.2	30.8
Dock4 Het	15.1 (±4.1)	31.7	59.5	8.7

b

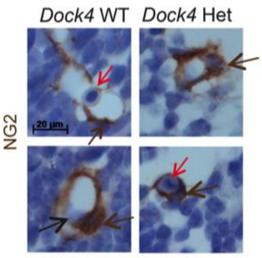

c

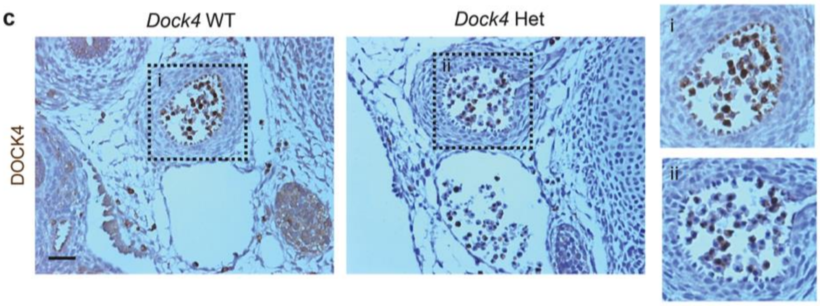

Supplementary Figure 9. DOCK4 controls blood vessel lumen calibre during embryonic development. (**a**) Images of sections immunostained with CD31 to visualize blood vessel lumens in the brain of E13.5 *Dock4* wild type (WT) and *Dock4* heterozygous (Het) embryos. Scale bar, 50 μm. *i-iv*: magnifications of areas outlined in larger images. Red arrows point to blood cells in small calibre lumens. Table shows analysis of lumen size (three embryos for each condition) in microscopic fields across the whole hemisphere. 40 vessels with largest diameter lumens were analysed for each embryo. (**b**) Images show pericyte coverage of blood vessels with different lumen sizes in the brain of E13.5 *Dock4* wild type (WT) and *Dock4* heterozygous (Het) embryos as detected by NG2 staining. Brown arrows point to NG2 positive pericytes; black arrows point to endothelial cells; red arrows point to blood cells in lumens. (**c**) Images of aortas of E13.5 *Dock4* WT and Het embryos, immunostained for DOCK4. Scale bar, 50 μm. *i-ii*: magnifications of areas indicated with boxes in larger images.

a

**siRNA**	**Target sequence or catalogue number**
Rac1 SMARTpool	1- CGGCACCACUGUCCCAACA<br>2- UAAAGACACGAUCGAGAAA<br>3- UAAGGAGAUUGGUGCUGUA<br>4- AGACGGAGCUGUAGGUAAA
RhoG SMARTpool	1- CUACACAACUAACGCUUUC<br>2- CCAGUCCGCCGUCCUAUGA<br>3- GCAACAGGAUGGUGUCAAG<br>4- CGUCAUCUGUUUCUCCAUU
RhoG ON-TARGETplus	5- CUACACAACUAACGCUUUC<br>7- GCUGUGCGCUACCUCGAAU
Cdc42 SMARTpool	1- GGAGAACCAUAUACUCUUG<br>2- GAUUACGACCGCUGAGUUA<br>3- GAUGACCCCUCUACUAUUG<br>4- CGGAAUAUGUACCGACUGU
Cdc42 ON-TARGETplus	5- ACAGTCGGTACATATTCCGTT<br>6- CAATCATAACTGTGACTGCTT<br>7- CAATAGTAGAGGGGTCATCTT
Dock4 SMARTpool	1- CAAAGGGUCUGGAGCAUUA<br>2- UAAGAGAGCUGAUGCUUGA<br>3- GGAAAUAGAUGUGAUAGUG<br>4- CCUCUUAUAUGACGAGCUA
Dock4 ON-TARGETplus	11- GGAGAAAAUUGCACGAUUA
SGEF SMARTpool	1- GAGAUGAUGUACACAAUUA<br>2- GAAGGAAGUUAGCAAGUUG<br>3- CAAAUGGCCUUGCCGCUAA<br>4- GAAAGGACAAGGAACAUUU
SGEF ON-TARGETplus	5- CAAAUGGCCUUGCCGCUAA<br>6- UUGGAGAUCUUGAUACGAA
Trio SMARTpool	Dharmacon: M-005047-00-0005
FLJ10665 SMARTpool	Dharmacon: M-020318-01-0005
Dock9 SMARTpool	1- GUAAACGAACGUCUGAUUA<br>2- ACUCAGAAGUUUCGAGAUA<br>3- CAGCUUGACUACUCAUUAA<br>4- CGGCAUUGCUUCUCCAUAU
Dock9 ON-TARGETplus	10- UCUGGAAAGCCGAGCGCUA<br>11- GCACAAUGGUUCACGGGAU
zyxin SMARTpool	1- GACAAGAACUUCCACAUGA<br>2- GAAUGUGGCUGUCAACGAA<br>3- GACCAAGAAUGAUCCUUUC<br>4- GGUGAGCAGUAUUGAUUUG
Non targeting	Allstars (Qiagen; Catalogue number #1027281)

Lentiviral shRNAs:

Rac1 shRNA1	CCCTACTGTCTTTGACAATTAT
Rac1 shRNA2	ACAGCTGGACAAGAAGATT
Dock4 shRNA1	CTCAGTATTTGCAGATATA
Dock4 shRNA2	CGCAAGGTCTCTCAGTTAT
Non silencing pGIPZ	ATCTCGCTTGGGCGAGAGTAAG

Retroviral shRNAs:

Dock4 retro shRNA1	CAGTTTCCCTCCAACTGTT
Dock4 retro shRNA2	CGTGAGCTCTTCAACAGTA
Nonsilecing pSMC	ATCTCGCTTGGGCGAGAGTAAG

b

<u>Sequence of DOCK4 SH3 domain used for expression of GST-fusion protein</u>

>DOCK4 SH3

ATGAGCACTTTTAAAGTTCTGCTATGTGGCGCGGTATTATCCCGTGTTGACG
CCGGGCAAGAGCAACTCGGTCGCCGCATACACTATTCTCAGAATGACTTGG
TTGAGTACTCACCAGTCACAGAAAAGCATCTTACGGATGGCATGACAGTAAG
AGAATTATGCAGTGCTGCCATAACCATGAGTGATAACACTGCGGCCAACTTA
CTTCTGACAACGATCGGAGGACCGAAGGAGCTAACCGCTTTTTTGCACAAC
ATGGGGGATCATGTAACTCGCCTTGATCGTTGGGAACCGGAGCTGAATGAA
GCCATACCAAACGACGAGCGTGACACCACGATGCCTGCAGCAATGGCAACA
ACGTTGCGCAAACTATTAACTGGCGAACTACTTACTCTAGCTTCCCGGCAAC
AATTAATAGACTGGATGGAGGCGGATAAAGTTGCAGGACCACTTCTGCGCT
CGGCCCTTCCGGCTGGCTGGTTTATTGCTGATAAATCTGGAGCCGGTGAGC
GTGGGTCTCGCGGTATCATTGCAGCACTGGGGCCAGATGGTAAGCCCTCC
CGTATCGTAGTTATCTACACGACGGGGAGTCAGGCAACTATGGATGAACGA
AATAGACAGATCGCTGAGATAGGTGCCTCACTGATTAAGCATTGGTAA

Supplementary Figure 10. Sequences of oligonucleotide duplexes and constructs. (**a**) Sequences siGENOME SMARTpool and individual ON-TARGETplus oligonucleotide duplexes used in the study (sense sequences shown). (**b**) Sequence of DOCK4 SH3 domain used for expression of GST-fusion protein.

Supplementary Figure 11

Figure 2a

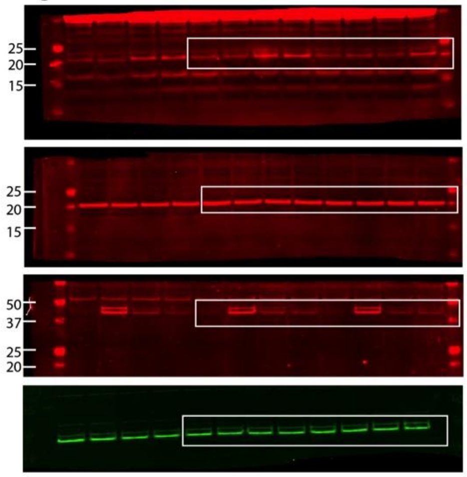

Figure 3g

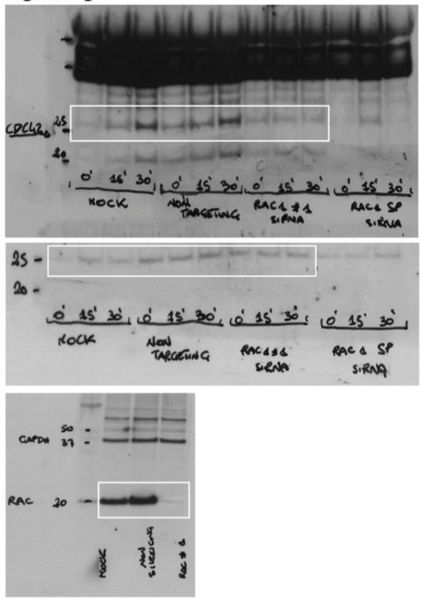

Figure 4a

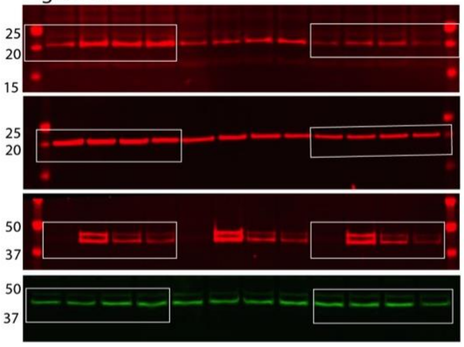

Figure 4b

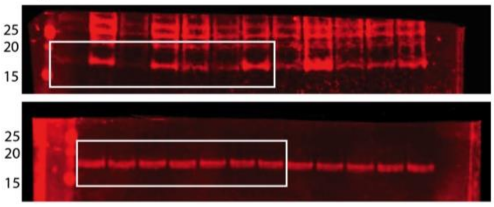

Figure 5a

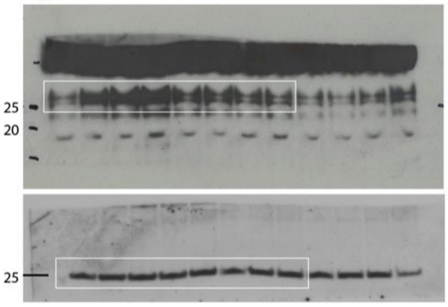

Figure 4d

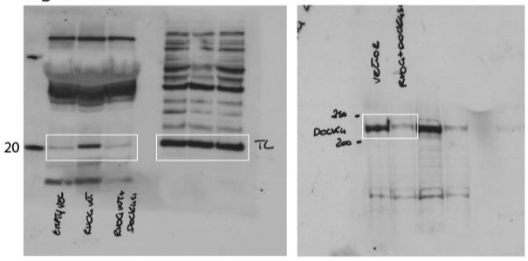

Figure 5b

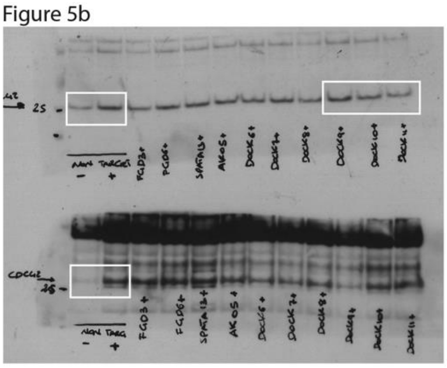

Figure 5d

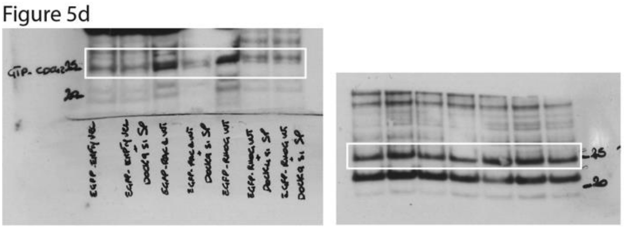

Figure 6b

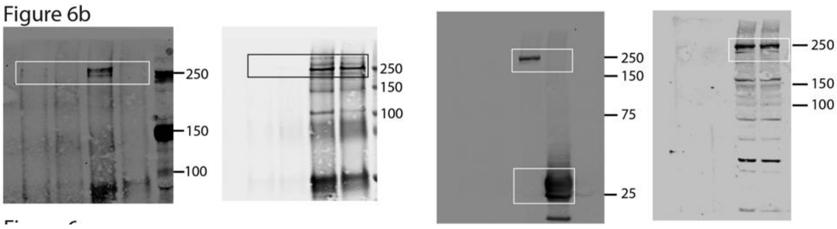

Supplementary Figure 11

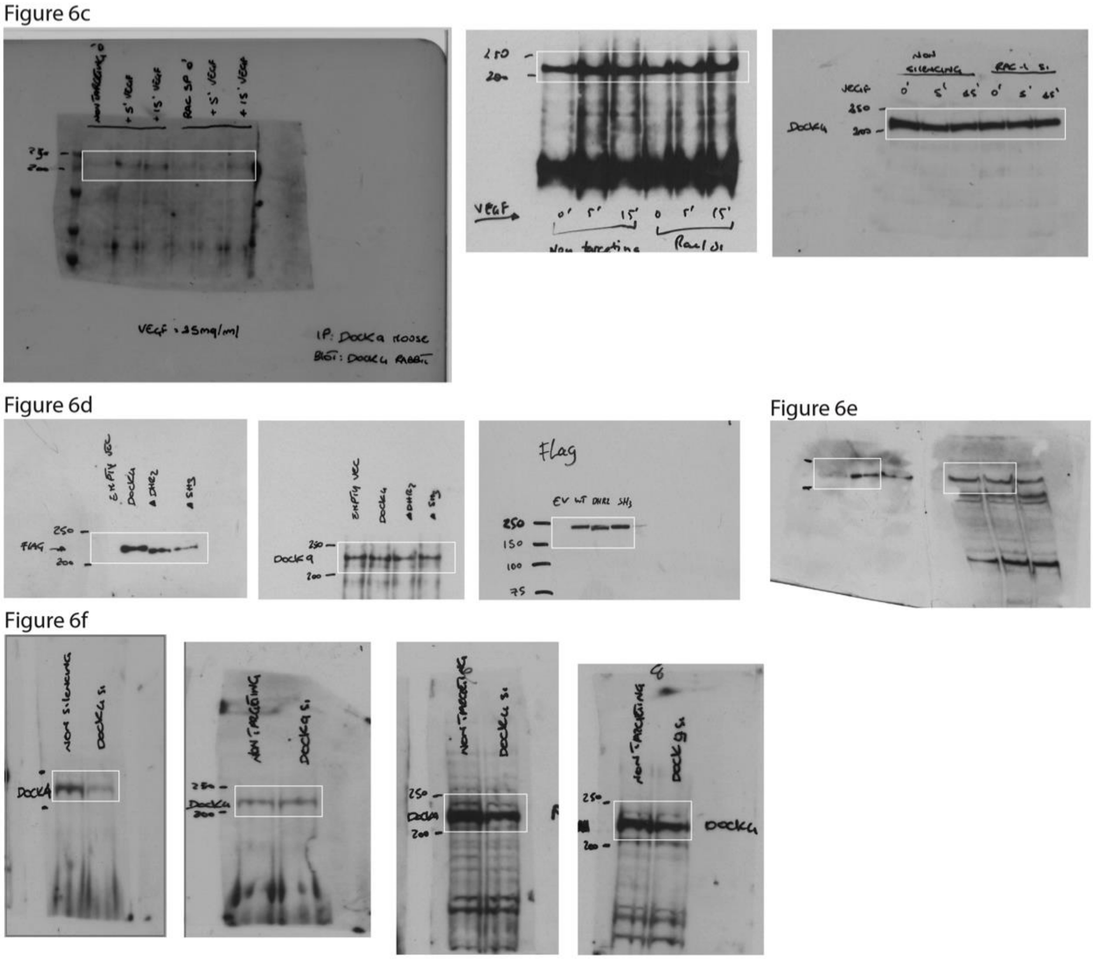

Supplementary Figure 11. Uncropped scans of key western blots in main figures. Boxes mark lanes depicted in figures.

GEF	Tubule morphology
FGD3	short & unbranched, rounded cells
ARHGEF18	short & unbranched, rounded cells
SOS1	short & unbranched
SOS2	short & unbranched
Trio	less tubules
RGNEF	clusters
SPATA13	clusters, rounded cells
SGEF	long & unbranched
FLJ10665	less tubules
FARP2	clusters
AKO57416	less tubules
FLJ10357	less tubules
Trad	less tubules, rounded cells
DOCK180	less branches or less tubules
DOCK2	less tubules
DOCK3	less branches or less tubules
DOCK4	less branches
DOCK5	short & unbranched
DOCK6	clusters
DOCK7	short & unbranched
DOCK8	short & unbranched
DOCK9	less branches

Supplementary Table 1. Rho GEFs required for angiogenesis. Tubule morphology in organotypic cocultures following knockdown of individual GEFs[22] in HUVEC at 5d after seeding onto CFs. Table shows GEFs with phenotypes observed over two or more independent experiments.

Supplementary Table 2

	Total tubule length (% mock)	*P*	Tubules (% mock)	*P*	Junctions (% mock)	*P*	Knockdown (% mock)
Non targeting	90.6		86.9		88.1		
Cdc42	26.7	**	39.7	**	16.1	***	70.4
RhoQ	56.9	*	65.9	ns	49.0	ns	88.0
RhoV	52.3	ns	58.0	**	31.7	*	36.8
RhoU	68.1	ns	68.7	ns	55.5	ns	28.1
Rac1	53.2	**	48.8	**	19.9	***	80.0
Rac2	85.9	ns	78.5	ns	64.0	ns	82.0
Rac3	58.1	ns	59.8	ns	34.9	*	88.0
RhoG	52.7	*	55.4	*	33.0	**	85.0
RhoA	59.1	*	65.6	ns	46.2	**	85.2
RhoB	74.0	ns	75.4	ns	56.2	ns	77.0
RhoC	60.5	**	53.5	**	37.9	**	76.0
RhoF	73.3	ns	75.3	ns	63.7	ns	47.9
RhoD	60.5	ns	61.6	ns	44.2	*	n.d.

Supplementary Table 2. Rho GTPases required for angiogenesis. Tubule formation in organotypic cocultures following knockdown of Rho GTPases in HUVEC at 5d after seeding onto CFs. Values represent mean of multiple experiments: non targeting, RhoQ, RhoG, *n*=4; Cdc42, RhoV, RhoU, Rac1, Rac2, Rac3, RhoA, RhoB, RhoC, RhoF, RhoD, *n*=3; RhoV, *n*=2); 6 or more images were quantified for each coculture. **P*<0.05; ***P*<0.01; ****P*<0.001 by Student's *t* test compared to mock transfected; ND, not determined. RhoG, Rac1 and Cdc42 were validated using additional oligonucleotide duplexes (see Supplemental Figure S2). Note that RhoJ regulates tubule formation[13]; ns, not significant.

Supplementary Table 3

Gene ID	Gene Name	Protein ID	Total Peptide Number
23348	DOCK9	194239705	461
254225	RNF169	148839382	33
31	ACACA	38679960	30
139818	DOCK11	145699123	20
23269	MGA	256017159	19
440689	HIST2H2BF	66912162	12
3188	HNRNPH2	9624998	11
7791	ZYX	4508047	11
6628	SNRPB	4507125	9
9732	DOCK4	92091572	8
63916	ELMO2	19718769	7
9406	ZRANB2	42741682	7
1153	CIRBP	4502847	6
23524	SRRM2	118572613	5
144455	E2F7	145580626	5
136319	MTPN	21956645	5
552900	BOLA2	73622130	5
494115	RBMXL1	21361809	5
57662	CAMSAP3	122937255	5
23396	PIP5K1C	31317309	5
983	CDK1	4502709	5
23396	PIP5K1C	31317309	5
23112	TNRC6B	148491080	4
55183	RIF1	56676335	4
57606	SLAIN2	149588928	4
5209	PFKFB3	4758900	4
57532	NUFIP2	32698730	4
5202	PFDN2	12408675	4
10625	IVNS1ABP	24475847	4
28971	C11orf67	34147384	4
6625	SNRNP70	29568103	3
84081	NSRP1	14149807	3
51479	ANKFY1	110815813	3
79596	RNF219	88759348	3
10523	CHERP	119226260	3
57396	CLK4	10190706	3
284695	ZNF326	33946297	3
26292	MYCBP	57242777	3
58525	WIZ	151301215	3
4131	MAP1B	153945728	3
9589	WTAP	21361159	3
23155	CLCC1	13194195	3

Supplementary Table 3. DOCK9 interaction partners identified by mass spectrometry. Number of peptide spectra are shown with Mascot score in parentheses. None of these proteins were identified in control IPs with 3xFLAG-mCherry.
